# Network, Anatomical, and Non-Imaging Measures for the Prediction of ADHD Diagnosis in Individual Subjects

**DOI:** 10.3389/fnsys.2012.00078

**Published:** 2012-12-21

**Authors:** Jason W. Bohland, Sara Saperstein, Francisco Pereira, Jérémy Rapin, Leo Grady

**Affiliations:** ^1^Department of Health Sciences, Boston UniversityBoston, MA, USA; ^2^Graduate Program for Neuroscience, Boston UniversityBoston, MA, USA; ^3^Siemens Corporation, Corporate Research and TechnologyPrinceton, NJ, USA

**Keywords:** ADHD, fMRI, network analysis, functional connectivity, resting state, machine learning

## Abstract

Brain imaging methods have long held promise as diagnostic aids for neuropsychiatric conditions with complex behavioral phenotypes such as Attention-Deficit/Hyperactivity Disorder. This promise has largely been unrealized, at least partly due to the heterogeneity of clinical populations and the small sample size of many studies. A large, multi-center dataset provided by the ADHD-200 Consortium affords new opportunities to test methods for individual diagnosis based on MRI-observable structural brain attributes and functional interactions observable from resting-state fMRI. In this study, we systematically calculated a large set of standard and new quantitative markers from individual subject datasets. These features (>12,000 per subject) consisted of local anatomical attributes such as cortical thickness and structure volumes, and both local and global resting-state network measures. Three methods were used to compute graphs representing interdependencies between activations in different brain areas, and a full set of network features was derived from each. Of these, features derived from the inverse of the time series covariance matrix, under an L1-norm regularization penalty, proved most powerful. Anatomical and network feature sets were used individually, and combined with non-imaging phenotypic features from each subject. Machine learning algorithms were used to rank attributes, and performance was assessed under cross-validation and on a separate test set of 168 subjects for a variety of feature set combinations. While non-imaging features gave highest performance in cross-validation, the addition of imaging features in sufficient numbers led to improved generalization to new data. Stratification by gender also proved to be a fruitful strategy to improve classifier performance. We describe the overall approach used, compare the predictive power of different classes of features, and describe the most impactful features in relation to the current literature.

## Introduction

Attention-Deficit/Hyperactivity Disorder (ADHD) is a complex developmental neuropsychiatric disorder characterized by abnormal inattentiveness, impulsivity, and hyperactivity. Recent estimates based on meta-analyses from the literature suggest a worldwide prevalence rate of ∼5.29 ± 0.28% in children 18 years of age or younger (Polanczyk et al., 2007), making it among the most common childhood disorders. Many children diagnosed with ADHD continue to exhibit symptoms throughout adulthood. The Diagnostic and Statistical Manual-IV Text Revision (DSM-IV-TR) describes three different types of ADHD: a *predominantly inattentive* type, a fairly uncommon *predominantly hyperactive-impulsive* type, and a most common *combined type* that includes features from each of the other two types (American Psychiatric Association, 2000). The biology of ADHD, including its genetics (Faraone et al., 2005; Banaschewski et al., 2010) and neurobiology (Tripp and Wickens, 2009), has received considerable attention but remains relatively poorly understood (see, e.g., Casey et al., 2007; Bush, 2010).

*Diagnosis*: There is no single, standard test for ADHD in children, and thus diagnosis requires the extended involvement of mental health professionals to accurately assess the existence and range of behavioral evidence and to differentiate ADHD from other disorders with overlapping symptomatology or from typically occurring behaviors. This process is costly and time-consuming. The use of non-invasive brain imaging methods coupled with advanced image analytics techniques holds the promise of great benefit for expediting or adding certainty to this diagnostic process. While this hope exists for essentially all neuropsychiatric disorders which rely on behavioral evidence for diagnosis, algorithms for objective classification of patients may hold special value in ADHD due to its heterogeneity, high prevalence, and particularly controversial diagnosis (Wolraich, 1999).

*Gender and IQ differences*: A number of demographic factors appear to be related to positive diagnosis of ADHD and may be useful in informing diagnostic algorithms. ADHD is diagnosed at a significantly higher rate in boys than in girls (Polanczyk et al., 2007). In 2007 in the United States, based on parent reports of any ADHD diagnosis in children ages 4–17, ADHD had been diagnosed in 13.2% of boys compared with 5.6% of girls^1^. Further, multiple studies have reported gender differences in the symptom profiles of children with ADHD (Gaub and Carlson, 1997; Newcorn et al., 2001; Gershon and Gershon, 2002), suggesting possible sex-specific mechanisms or manifestations of the pathophysiology of the disorder. Cognitive measures including Full Scale IQ as well as Verbal and Performance IQ are also reliably different between individuals with ADHD and typically developing controls (TDCs; Frazier et al., 2004).

*Neuroimaging correlates of ADHD*: Family and twin studies of ADHD have established high degrees of heritability (Faraone et al., 2005; Burt, 2009), supporting the existence of a biological and genetic basis for the disorder. Brain imaging may then be viewed as a method for providing quantitative or semi-quantitative *endophenotypes* (Doyle et al., 2005), measures which are theoretically more closely related to the underlying biological etiology than are the behavioral signs and symptoms. To this end, a wide range of anatomical and functional brain imaging studies have been conducted comparing children with ADHD to typically developing children, and have described a number of relatively consistent results (Giedd et al., 2001; Durston, 2003; Bush, 2010). These range from gross findings that total cerebral volume may be reduced by ∼3–4% (Valera et al., 2007) and that global cerebral glucose metabolism is substantially reduced (Zametkin et al., 1990), to results demonstrating reduced cortical thickness in the right superior frontal gyrus across the lifespan (Almeida et al., 2010), and numerous reports of altered anatomical or functional connectivity (Konrad and Eickhoff, 2010; Liston et al., 2011) in individuals with ADHD relative to controls. The breadth of the available functional and structural imaging studies, which are too numerous to review here, have generally implicated prefrontal cortex (including dorsolateral and ventrolateral prefrontal areas), anterior cingulate cortex, parietal cortex, striatum, and cerebellum.

Despite the promise of brain imaging for aiding clinical diagnosis, currently no imaging techniques are recommended for this purpose (Bush, 2010). One possible explanation for this unfulfilled promise could be that the measures necessary for accurate diagnosis may be high-dimensional and not readily observable from classical univariate image analysis methods. Further, studies conducted in small samples may not sufficiently generalize to larger populations.

*Complex brain networks*: It is long established that alterations in inter-regional neuronal connectivity, as in the case of so-called *disconnection syndromes*, can underlie complex brain disorders (Geschwind, 1965). Recent theories of the basis for neuropsychiatric disorders have reinvigorated these conceptualizations (e.g., Mega and Cummings, 1994; Tekin and Cummings, 2002; Geschwind and Levitt, 2007). Concomitant with such theories, advances in brain imaging and data analytic methods (as well as the rise of the more general domain of systems biology), have enabled the generation and quantitative analysis of *complex brain networks* built from structural and/or functional imaging data (Bullmore and Sporns, 2009). Such networks, including those constructed based on *resting-state fMRI (rs-fMRI)*, are now commonly used in the study of normal and abnormal cognitive function.

Resting-state fMRI, based on low-frequency BOLD signal fluctuations that occur while the subject is resting and performing no explicit task, has garnered significant recent interest as a tool for finding clinically relevant biomarkers and/or for measuring responses to treatment (Greicius, 2008). Networks built from correlations or related measures calculated across instances of functional imaging time series obtained during rest may be interpreted, at least partly, to reflect the intrinsic *functional connectivity* between different brain areas, and their properties may be relevant for understanding typical and atypical variability across the population. A number of studies have provided sparse evidence for altered connectivity in ADHD, but much further work is necessary to fully characterize network phenotypes as well as inter-subject variability (Castellanos et al., 2009).

Currently a large number of methods exist for the construction of functional connectivity networks. Simulation studies performed by Smith et al. (2011) have demonstrated direct evidence that not all methods are equivalent in their ability to estimate the existence of underlying inter-regional connections. Thus the potential for discovering network-based measures that correlate with the presence or absence of ADHD may hinge on the methods used to define each individual network’s elements, including nodes, presence/absence of edges between node pairs, and any weights assigned to those edges.

*Network analytics*: The *structure* of a system (physical or biological) is an abstract concept that can be difficult to quantify in a manner that can be used to predict its characteristics or to distinguish between different types of systems. However, by representing the structure of a system with a network model it becomes possible to quantify various measurements of the network that may be used to characterize the system. For example, a series of network measures has been used to determine whether the configuration of a network of cells derived from histological section images can predict the presence of cancer (Gunduz et al., 2004; Demir et al., 2005; Khurd et al., 2011; Chekkoury et al., 2012). Historically, these network measures have been widely used in chemical graph theory for a very long time (e.g., Wiener, 1947) to predict various structural and behavioral properties of molecules (for reviews of this type of usage see Hansen and Jurs, 1988; Bonchev and Rouvray, 1991; Mihalic and Trinajstic, 1992). More recently, a wide variety of measures have been explored and used for the structural quantification of systems spanning scientific disciplines (see Costa et al., 2007). Furthermore, network-level properties such as graph efficiency (Latora and Marchiori, 2001) have already been demonstrated to be useful markers of ADHD (Wang et al., 2009).

### ADHD challenge

In this paper we describe our efforts to use attributes derived from MR images as well as non-imaging phenotypic measures to predict the presence or absence of an ADHD diagnosis in child and adolescent subjects. This opportunity was made possible by the availability of a large dataset comprising structural and resting-state functional MRI scans and associated non-imaging phenotypic data (e.g., gender, age, and cognitive testing measures) from 776 children and young adults. These data were provided by the ADHD-200 Consortium, a “self-organized, grassroots initiative, dedicated to accelerating the scientific community’s understanding of the neural basis of ADHD through the implementation of discovery-based science” as part of the *ADHD-200 Global Competition*^2^. This competition invited researchers from all disciplines to participate in an effort to produce the highest performance imaging-based diagnostic classification algorithm for ADHD. Scoring was based on a pre-specified point system that involved both base diagnosis and diagnosis of ADHD subtype. A separate award was provided for the most innovative neuroscientific examination of ADHD. Our group finished fifth overall in the classification competition, and the present paper describes our approach and continued efforts to improve and characterize classification methods and results.

The open availability of large *N* datasets with compatible, commonly coded primary data and metadata is critical to successfully fulfilling the promise of exploratory and machine learning approaches for the discovery of principles of normal and disordered brain function (Biswal et al., 2010; Milham, 2012). The ADHD-200 sample represents a starting point for this approach in ADHD research and presents a test bed for utilizing large sets of anatomical, network, and non-imaging measures for objective diagnosis of complex neurobehavioral disorders that currently require extensive, continued behavioral testing for diagnosis, and lack clear biomarkers.

### Approach

**Figure 1:**
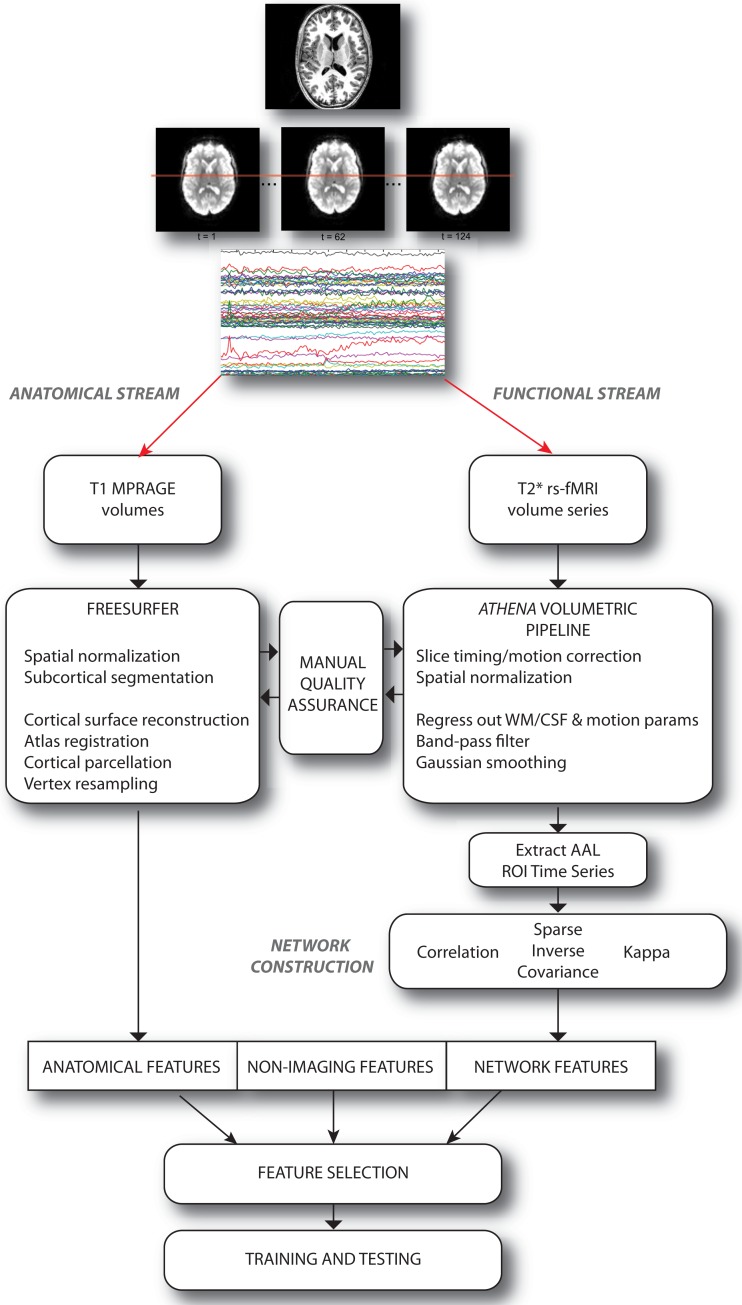
**Schematic describing the overall pipeline through which data were processed and submitted to machine learning algorithms for classification**. Note that anatomical and functional/network streams were largely independent.

We approached the diagnosis problem by examining the predictive power of three sets of features or attributes: (i) non-imaging phenotypic features, (ii) anatomical features derived from structural brain images, and (iii) network features derived from graphs depicting functional connectivity during rs-fMRI. A set of over 12,000 features was computed for each individual subject; these features were provided, in the groups described above, to train classifiers. These classifiers were evaluated using a cross-validation approach, and then used to predict the presence or absence of ADHD in a separate group of test subjects. The multi-stage pipeline that was used to perform these analyses is schematized in Figure 1 and described in detail below.

Prediction using the non-imaging phenotypic features (e.g., age, gender, and IQ) provides a baseline performance level which, in the current dataset, is well above chance. We anticipated that the addition of certain anatomical and network features would add predictive power, increasing overall performance of the classifiers. Based on previously observed gender differences, we also anticipated that *separate classifiers* may be needed to maximize predictive power for boys vs. girls. Below we describe the overall results of classification using this approach and discuss the power of the different classes of features.

## Materials and Methods

### ADHD-200 dataset

Anatomical and resting-state functional MRI scans were performed at 8 different facilities on children and young adults ages 7–21 years (mean: ∼12 years), approximately half (∼53%) male. Participants were diagnosed as either typically developing or ADHD-Hyperactive, ADHD-Inattentive, or ADHD-combined type. These data and various metadata describing subject phenotypic traits (including diagnosis) were made available to download through the ADHD-200 Consortium. Too few cases of ADHD-Hyperactive type were available for any practical analysis and, in general, the other two subtypes are not distinguished in our analysis below except to assess subtype-specific performance. In other words, we describe classifiers built to determine the presence or absence of ADHD irrespective of subtype, but we were able to analyze *post hoc* whether their performance differed depending on the subtype. While the ADHD Global Competition included scoring based on subtype diagnosis, and while being able to differentiate subgroups within a diagnostic category is of high potential clinical value, we chose to focus efforts here on the problem of *primary* diagnosis, applying a large and diverse feature set, combined with gender-stratified training that limited the number of training examples that would be available for each individual subtype.

Imaging parameters (i.e., repetition time, number of volumes acquired, and other MR acquisition parameters) differed somewhat across sites^3^, and some subjects were imaged more than once. For subjects with multiple rs-fMRI sessions, time series were concatenated after preprocessing.

Non-imaging phenotypic attributes that were included in the dataset and used in the classifiers included:

(1) Age
(2) Gender
(3) Handedness
(4) Verbal IQ
(5) Performance IQ

### Overall machine learning framework

Our approach to diagnosing Attention-Deficit/Hyperactivity Disorder combined the use of anatomical markers, non-imaging phenotypic data (above), and network analytics computed from graphs constructed from each individual’s resting-state fMRI data. We calculated a variety of standard and new quantitative markers and applied machine learning algorithms to perform the ADHD classification.

The anatomical and network features (described below) were normalized to have zero mean and unit standard deviation (SD) across all subjects in the dataset. Any features with constant values (across subjects) were excluded at this stage. The non-imaging phenotypic features were used without any normalization, and missing values of Verbal or Performance IQ were replaced by the respective population average. We augmented these with several binary features: *NoIQ* (1 if the subject was missing IQ scores, 0 otherwise) and *Site1*–*Site8* (1 if the subject was imaged at that site, 0 otherwise).

We performed a two-fold cross-validation procedure over the released training data. Examples (subjects) were sorted by site, classification label (i.e., diagnosis), gender, and age, and were assigned in round-robin fashion to folds 1 and 2; this was done to ensure that each fold contained equal proportions of examples with similar values for those attributes. Next the dataset was separated by gender (474 boys and 280 girls were available in the final training data), and all the steps described below were performed separately for the two resulting datasets, with results combined at the end. The assessments of overall diagnostic performance were made after pooling all individual classification results across the gender groups (i.e., treating the outputs as if they had come from a single classifier).

We used three methods to rank features (Guyon, 2003), and used the resulting rankings to select between 5 and 6000 (or *all* in the cases where more than 6000 features were available) features using each method, with the selection procedures performed inside the training data of each cross-validation fold. Two filter methods were used to score each feature individually: (i) a 2 sample *t*-test (comparing the feature values for ADHD participants vs. controls) and (ii) the accuracy of a classifier trained and tested in nested cross-validation over that single feature. We also applied a wrapper method, recursive feature elimination, which also made use of nested cross-validation. This method consisted of training a linear support vector machine classifier on all available features, then scoring features by the magnitude of the weights assigned to them. The bottom 50% features were then eliminated from consideration and the procedure repeated until there were 10 or fewer features. Final feature scores were a combination of the last round in which each feature survived and, within that, the magnitude of the weight assigned by the classifier, such that the last surviving features had the higher scores.

The three feature selection methods were parallel equivalents of one another (i.e., one replaces the other, each resulting in a *ranking* of the overall feature set). Because features were ranked using each of the above methods throughout a cross-validation process, we computed the average rank of each feature (across folds) for each method. These average rankings were then used for selecting the features used to train classifiers using examples from the *entire* training dataset. Specifically, we chose the top *K* ranked features for a variety of values of *K*. Performance was then assessed using the *separate test set* released by the ADHD-Consortium, which was not used for either training or feature selection. A linear SVM classifier (LIBSVM; Chang and Lin, 2011) with regularization parameter λ = 1 was used as the classification algorithm with all sets of selected features; other classification algorithms were tested in preliminary studies, but provided similar or inferior overall performance.

#### Evaluating classification performance

Comparing the value of different classifiers requires a measure capable of representing the utility of one classifier over another. One natural measure is the *accuracy* which quantifies the probability that the classifier will make a correct prediction of ADHD vs. TDC. However, under differing practical scenarios, it may be more important to be confident that the classifier provides a correct diagnosis of ADHD positive (high true positive rate) or that the classifier provides confidence in ruling out an ADHD-positive diagnosis (low false positive rate). A mechanism for characterizing the value of a classifier under such different scenarios is the *Receiver Operating Characteristic* (ROC) curve, which plots the probability of predicting a true ADHD positive given a tolerance for a certain percentage of false ADHD-positive results. Consequently the *area under the ROC curve* (AUC) can be used to measure the value of one classifier compared to another, regardless of the practical scenario. An AUC of 1.0 indicates a perfect classifier (i.e., a true positive is always obtained without sacrificing any false positives) while an AUC of 0.5 indicates that the classifier does no better than chance in predicting the presence/absence of ADHD. In the present study, all results are reported using this measure and are provided for the cross-validation stage (on the non-training folds) as well as for the separate test stage.

In order to compute the ROC curve for a classifier, we ranked the examples by the magnitude of the LIBSVM decision value output for each. This was obtained for each example by multiplying the weight assigned to each feature by the value it took in that example and adding over features. We then computed the true positive and false positive rates obtained when setting the classification threshold at each point in the ranking. For the set of results using all feature types, we also provide accuracy scores (which represent one point on the ROC curve that maximizes the overall percent correct in binary diagnosis).

### Anatomical features

All structural MRI scans (T1-weighted MPRAGE volumes, anonymized using a “defacing” algorithm to protect patient confidentiality) were processed through the FreeSurfer software package^4^, version 5.0.0 using the typical “recon-all” procedures. Specifically, this software was used to perform intensity normalization, skull stripping, white matter segmentation, and tessellation and reconstruction of the cortical surface in each hemisphere. In addition, individual surfaces were registered to a spherical atlas space, and the cortex was parcellated into macro-anatomical regions. Furthermore, from the T1 volumes, a set of subcortical structures were segmented, and a variety of morphometric measures were estimated. Technical details of these procedures are described elsewhere (Dale et al., 1999; Fischl et al., 1999a,b). The quality of MR images, Talairach registration, pial surface demarcation, and surface inflation were assessed using a manual inspection protocol. Approximately 2% (14 of 776) of the images failed this stage of quality assurance and were removed from the subsequent analyses. Cortical surface-based features (thickness and curvature) were computed for each subject and resampled onto an icosahedral surface model defined in the atlas space. This surface consists of 2,562 locations (vertices) in each hemisphere, equally spaced around the inflated sphere. Based on initial experiments, we discarded average curvature features and focused on local thickness features as possible ADHD diagnostic aids. Thus, for each subject we calculated a total of 5,124 local cortical thickness features.

Additionally, automated surface-based cortical parcellations and volume-based subcortical structure segmentations were computed for each subject (Fischl et al., 2002, 2004), and a series of statistics were calculated for the individual structures (average cortical thickness, surface area, volume, mean curvature, and SD of these measures for each cortical region-of-interest). The volumes of various subcortical gray and white matter structures were also estimated, and normalized by each individual’s total intracranial volume (ICV) to help control for age effects. FreeSurfer also calculated the volumes of subcortical areas with hypointensities in gray or white matter; these were also normalized by ICV and included in the overall feature set.

### Network features

#### Pre-processing rs-fMRI data

Individual subject resting-state functional connectivity networks were generated (using three different network construction methods, see below) from pre-processed rs-fMRI time series data, and a large set of network measures were calculated from these networks. Functional MRI preprocessing relied on scripts provided publicly by the NeuroBureau^5^, specifically using the so-called *Athena Pipeline*. All raw rs-fMRI data were reprocessed using these scripts, adapted to our local computing environment, which used methods from the publicly available AFNI (Cox and Hyde, 1997) and FSL (Smith et al., 2004) software packages. This pipeline^6^ included steps for normalization of anatomical volumes to an age-specific (4.5–18.5 years) template brain volume in MNI-space (Fonov et al., 2011; contrast with surface-based registration in our Freesurfer-based anatomical pipeline) using a low-dimensional non-linear deformation, and realignment and co-registration of functional images to this space. The first 4 EPI volumes in each rs-fMRI scan were discarded due to T1-equilibration effects. Slice timing correction was performed, and the mean activation time courses from white matter (WM) and cerebrospinal fluid (CSF) as well as estimated motion parameters and a set of low-order polynomials were used as nuisance regressors. Resulting voxel-wise time courses were band pass filtered (0.009 Hz < *f* < 0.08 Hz) according to common practice in rs-fMRI analysis (Cordes et al., 2001). Region-specific average time courses were extracted from each subject’s data using the Automated Anatomical Labeling (AAL) template atlas (Tzourio-Mazoyer et al., 2002), which consists of 116 brain regions-of-interest (ROIs) demarcated in MNI-space based on sulcal and gyral landmarks in the MNI single-subject template atlas.

#### Network construction

Several different methods have been proposed for inferring functional connectivity from a resting-state time series acquisition. Smith et al. (2011) used simulation studies to test a wide variety of methods for inferring connections from fMRI time series; following these results, we deployed three of the best-performing methods for estimating weighted networks from the AAL time course data above:

(1) Correlation, with correction for temporal autocorrelation (*Corr*)
(2) Sparse regularized Inverse Covariance (*SIC*)
(3) Patel’s Kappa (*Kappa*)

*Corr* networks were based on calculations of Pearson correlation coefficients between the average time series for pairs of AAL regions. These were then converted into *P*-values under the null hypothesis of no correlation using a Fisher transformation, and taking into account temporal autocorrelation. False-discovery rate (Benjamini and Hochberg, 1995) was used to correct for multiple comparisons at a rate of 0.01. Edges representing significant correlation between nodes (AAL regions) were assigned weights equal to the corresponding pairwise correlation coefficient; edges for which the corrected correlations were not significant were set to zero.

The *SIC* networks were created using methods from the Sparse Learning with Efficient Projections (SLEP) toolbox (Liu et al., 2009). In particular, the inverse of the AAL time series covariance matrix was computed under an L1-norm regularization penalty (see also Friedman et al., 2008; Huang et al., 2010), yielding a measure of partial correlation. Based on Smith et al. (2011) and exploratory testing, we chose a regularization parameter of λ = 0.1 in all cases. L1-regularization enforces sparsity in the inverse estimate, and thus these networks contained many edges with weight values that were close to zero. Non-zero edge weights were real valued, between 0 and 1.

*Kappa* networks were computed based on the κ measure described in Patel et al. (2006), extended to continuous (non-binary) measurements as described in Smith et al. (2011). This is a measure of connection strength based on conditional states of pairs of normalized time series. *Kappa* networks had continuous-valued edge weights and were not subjected to an edge threshold.

In definitions below we refer to a graph *G* = {*V*, *E*} consisting of a set of vertices (or nodes) *V*, and edges *E*. We denote an individual *i*th vertex as *v_i_* ∈ *V* and an edge spanning vertex *v_i_* and *v_j_* as *e_ij_* ∈ *E*. We denote the weight assigned to edge *e_ij_* as *w_ij_*. In all cases, inferred edges were weighted using real-values (i.e., they were not binarized, as is common in the literature). The *absolute values* of edge weights were used to calculate network measures. These edge weights computed in our network construction methods are *affinity weights*, which are larger if two nodes are more strongly connected. Therefore, in order to compute meaningful measures based on paths, it was important to convert the edge weights to *distance weights*, which are small if nodes are similar. The appropriate relationship between affinity and distance weights was given in Grady and Polimeni (2010) as:

(1) $$w_{\text{distance}}=\frac{1}{w_{\text{affinity}}},$$

which is the same relationship between resistance (distance) and conductance (affinity) in an electrical circuit; see Grady and Polimeni (2010) for more discussion of this point. In the following sections, we will specify whether the affinity or distance weights were used to compute each measure. All networks were *undirected* (*w_ij_* = *w_ji_*) and contained 116 nodes (corresponding to the regions of the AAL atlas) and 6,670 possible edge weights.

We subjected each network to a wide range of feature analysis in order to capture specific markers which might aid in predicting the presence/absence of ADHD. We examined a large number of standard measures from the resting-state network literature (see, e.g., Sporns, 2010). However, since it was unclear which network characterizations might provide insight into ADHD, we broadened the range of network features considered to include features derived from the literature outside of neuroscience, such as those reviewed in Costa et al. (2007) and Grady and Polimeni (2010). Specifically, in addition to examining the standard “scale-free” and “small-world” properties of the networks, we also looked for measures that quantify network connectivity (both single path and parallel path), network separability, network cycle structure, and the recurrence of certain network motifs.

#### Standard network measures

We calculated measures of graph connectivity that are derived from the literature on “scale-free” and “small-world” networks. To characterize small-world properties, we computed the average path length, diameter, radius, and mean/max/min clustering coefficients for each network (Grady and Polimeni, 2010). These measures would help us distinguish if the functional connectivity network of ADHD subjects more strongly resembled a small-world network (as first described in Watts and Strogatz, 1998). It has been suggested in the literature to also examine the related measures of global efficiency and the harmonic mean of the path lengths (Latora and Marchiori, 2001), which we have also included in our study.

Similarly, to examine the scale-free properties of the network, we computed the entropy of the degree structure and the assortativity (Newman, 2002). These computations could reveal if the functional connectivity networks of ADHD patients were more or less scale-free than typically developing controls. Since scale-free networks (in which the node degree distribution approximately follows a power law) are modeled through a process of preferential node attachment, a difference in the scale-free properties could suggest an underlying difference in the process of network/circuit formation for ADHD patients.

We also examined several conventional measures that were computed for each node. These features would reveal if the role of a particular node within the overall network were different for ADHD subjects. Specifically, for each node we computed its degree, betweenness, eccentricity, and central point dominance, providing a set of measures of how the node is situated in the overall network (Grady and Polimeni, 2010). The weighted degree of a node (region) can be interpreted as a measure of its overall functional connectivity with the rest of the brain, while betweenness has a possible interpretation as indicating the “importance” of a region in the overall flow of information. These per-node features were also condensed into a small set of measures characterizing the overall network such as mean/max/min degree, mean/max/min betweenness, and the entropy of the betweenness values. For the novice reader, a brief introduction to a set of network measures is provided in the Appendix.

All of these measures depend on shortest paths and were therefore computed using distance edge weights. Since the clustering coefficient measures are typically defined for unweighted graphs, we computed these coefficients by treating a connection between nodes of any weight as a connection (effectively setting all edge weights to unity).

#### Parallel connectivity measures

One criticism of the conventional measures described above is that they generally rely on measuring shortest paths between pairs of nodes in the network. However, information which spreads through multiple channels (such as a diffusion process) is more sensitive to the collection of strong *parallel* paths connecting a pair of nodes rather than the presence or absence of a strong *single* path connecting the pair. One way of measuring parallel paths is through the concept of *effective resistance*, which treats the network as a linear resistive circuit (Klein and Randic, 1993). Note that it is important to treat all edge weights as affinity weights in an electrical circuit interpretation (Grady and Polimeni, 2010).

To quantify the strength of parallel connections between each node and the rest of the network, we calculated the eccentricities of each node with respect to the resistance distance instead of the conventional shortest-path distance. These per-node eccentricities can also be used to quantify the overall parallel connectivity of the network via the radius and diameter of the effective resistance as well as the Kirchhoff Index used in chemical graph theory (Bonchev et al., 1994; Diudea and Gutman, 1998).

#### Network separability

Some networks have one strongly interconnected component while others have multiple different functional clusters. The separability (or modularity) of a network is not easily characterized by the previous measures which depend on examining strong paths between node pairs. To quantify the separability of a network, we computed two classical measures. Specifically, we calculated the Fiedler values of the unnormalized and normalized Laplacian matrix. The unnormalized Laplacian matrix is defined as *L* = *D* − *W* where *W* is the weighted adjacency matrix (with affinity edge weights) and *D* is a diagonal matrix of weighted node degrees such that *D_ii_* = ∑*_i_W_i_*. The normalized Laplacian matrix is defined as $\bar{L}=D^{-1}L.$ The Fiedler values are the second-smallest eigenvalues of these matrices, which are known to reflect the separability of the graph into two pieces (Fiedler, 1973 originally termed this value the *algebraic connectivity* to reflect this property). We also computed the third-smallest eigenvalue of these matrices as a feature, since this value reflects the separability of the network into three components.

A feature that describes separability of the graph is its *isoperimetric number* (Mohar, 1989), which describes the smallest ratio of

(2) $$\iota \left(S\right)=\frac{\text{cut}\left(S,\bar{S}\right)}{\left|S\right|},$$

for any node subset *S* ⊂ *V* such that 0 < |*S*| ≤ 1/2|*V*|, where

(3) $$\text{cut}\left(A,B\right)=\underset{e_{ij},\;\text{s}.\text{t}.v_{i}\in A,v_{j}\in B}{\sum }\;w_{ij}.$$

Similarly, a normalized isoperimetric number of a network is also defined as the minimum ratio

(4) $$\bar{\iota \left(S\right)}=\frac{\text{cut}\left(S,\bar{S}\right)}{\text{Vol}\left(S\right)},$$

for any node subset *S* ⊂ *V* such that 0 < Vol(*S*) ≤ 1/2Vol(*V*) where $\text{Vol}\left(S\right)=\sum_{v_{i}\in S}\;d_{i}.$ Unfortunately, calculation of either isoperimetric number for a graph is NP-Hard (Mohar, 1989). Therefore, we performed an estimation of the isoperimetric number using different clustering methods to find good candidate sets *S* and taking as features the values of *℩*(*S*) and $\bar{\iota \left(S\right)}$ that are smallest over all clustering methods. Specifically, we applied the spectral clustering and isoperimetric clustering algorithms (Grady and Schwartz, 2006) to estimate the isoperimetric number. Note that if the network is disconnected, the Fiedler values and isoperimetric numbers will all be zero. However, in our experiments with these data and set of network construction methods it was uncommon to find disconnected networks.

Other measures of modularity have been explored in the literature (e.g., the Q measure; Rubinov and Sporns, 2011) that characterize the goodness of a particular node partitioning in a network. However, these measures are dependent on the partitioning algorithm and, since these networks were generally quite dense, greedy algorithms (e.g., Newman, 2004) are unlikely to provide meaningful results. Due to the dependence on partitioning algorithm, these measures were not included as features in our study.

#### Cycle measures

The most common way to quantify network structure is from the standpoint of connectivity between various node pairs. Although these connectivity measures give an indication of the ability to pass signals between nodes, they fail to characterize the structure of feedback loops in the network. In fact, the cycles of complex networks have recently been shown to contain substantial information about certain types of networks (Khurd et al., 2011). Consequently, we followed Khurd et al. (2011) to produce a set of features characterizing the cyclic structure of these networks based on computing a minimum cycle basis for each network (Horton, 1987; Kavitha et al., 2008). All cycle lengths were computed using distance edge weights.

Most cycles in the minimal cycle basis are triangles (i.e., only three nodes and edges). Our quantification of the cycle structure was done by calculating the percentage of non-triangle cycles, their mean/max length and the sum of all cycle lengths.

#### Sparsity measures

Network sparsity gives some indication of the overall synchronization of the network and the overall energy expended by the network. A natural measure of sparsity is to count the number of non-zero edges in a network, but this measure *assumes that all nodes are valid*. Any connection between two ROIs, however, might potentially be subdivided into a string of smaller nodes. Similarly, any ROI might potentially be subdivided into a number of small, tightly coupled nodes. If this subdivision was accidentally made for one network but not for another, then by taking the number of non-zero edges as the sparsity measure, the subdivided network would appear more sparse (if it were a connection that was subdivided) or less sparse (if an ROI were subdivided) than the unsubdivided network.

To build a measure that is robust to the subdivision problem, we examined a sparsity measurement derived from linear algebra. Specifically, let *P* be a permutation matrix representing a node ordering in the network. Then, the sparsity of the Cholesky factor for the matrix $\tilde{L}=PLP^{T}$ may be compared to the sparsity of the original Laplacian matrix, *L*, to determine the amount of “fill-in” (new edges) created by the ordering. It has been shown (e.g., Grady and Polimeni, 2010) that Gaussian elimination of a node (row/column) in the Laplacian matrix creates a new reduced Laplacian matrix, representing a graph in which the eliminated node is removed, and a connection is created between all neighbors of the eliminated node. Consequently, if the removed node is part of a path then the two neighbors of the eliminated node are connected by a single edge in the reduced graph, causing no fill-in. Similarly, if the eliminated node is part of a fully connected clique, then no new edges are created in the reduced graph, since all of its neighbors were already connected. Therefore, we believe that by comparing the sparsity of the Laplacian matrix to the amount of fill-in created by the Cholesky decomposition of the reordered matrix, a measure of *intrinsic* sparsity may be obtained which is robust to subdivisions of connections into paths or ROIs into tightly connected clusters of many nodes.

The intrinsic sparsity that we have defined is substantially dependent on the node ordering that defines the permutation matrix *P*. Unfortunately, finding an ordering that produces a *minimum* fill-in is known to be an NP-Hard problem (Papadimitriou, 1976). Consequently, the field of numerical linear algebra has produced several ordering strategies that are known to provide low fill-in for different types of matrices/networks, such as the Approximate Minimum Degree (AMD), Cuthill–McKee, and Dulmage–Mendelsohn orderings. To quantify intrinsic sparsity of the networks, we used as network features the sparsity of the original Laplacian matrix and the fill-in obtained by the above orderings, as well as the fill-in produced by the lexicographic ordering as a reference. Since the sparsity measurements look only at the structure of the network, rather than the edge weights, we computed these sparsity features for each network when the affinity weights were thresholded at the levels of {0, 0.1, 0.3, 0.5, 0.7, 0.9}.

#### Network motifs

The previous features all examined structural properties of the networks in terms of paths between node pairs, node centrality, degree distribution, cycles, separability, and sparsity. However, it may be possible that what distinguishes ADHD subjects from TDC subjects is the presence/absence of a particular circuit of connections in the brain. Unfortunately, measuring the presence of all possible circuits in the network is combinatorically prohibitive, even for our networks of 116 nodes. For example, even the number of possible subgraphs of three nodes is ∼250,000, which is far too many to meaningfully explore without an enormous test dataset. Consequently, we examined the more tractable set of the 6670 possible 2-cliques (edges), using each affinity edge weight as a feature.

### Feature impact

For features of all types, *feature impact weights* were calculated after training the classifier on the complete training set (all folds). The linear SVM learns weights associated with each feature, and it is common to use the absolute value or squared value of these weights as measures of importance or impact in the classification problem. However, since features were normalized to have zero mean, it was important to consider the sign of feature values in assessing impact. Therefore we calculated feature impact for each class (ADHD vs. TDC) by multiplying the learned feature weight by the mean value of that feature *within that class*. The sign of the feature impact indicates whether those features were, on average, driving the classifier toward that class diagnosis (for positive impacts), or away from that diagnosis (negative impacts).

## Results

The results detailed below describe the predictive power of individual classes of features (non-imaging phenotypic, anatomical, and network features) as well as the potential for improving predictions by combining different feature classes. By grouping features in this way, we are able to examine the promise for different avenues of diagnostic aids (e.g., structural features vs. functional network features, which necessitate additional MR scan time). We describe a series of classifiers for each grouping of features, which were built using the multiple feature selection methods described above, with different numbers of selected features. All results describe both performance in cross-validation (testing on the fold not used for training) and on the separate test set (training on all folds of the cross-validation/training set). In nearly all cases, classifiers were able to predict the presence/absence of ADHD in individual subjects from the test set at above chance levels as measured by the area under the ROC curve. Unless otherwise specified, results are based on a dataset consisting of 755 training examples, which is 21 fewer than the 776 provided in the original data. These datasets were excluded due to processing or quality assurance problems in either our anatomical stream (14 subjects) or our network stream (7 additional subjects).

### Gender differences

**Figure 2:**
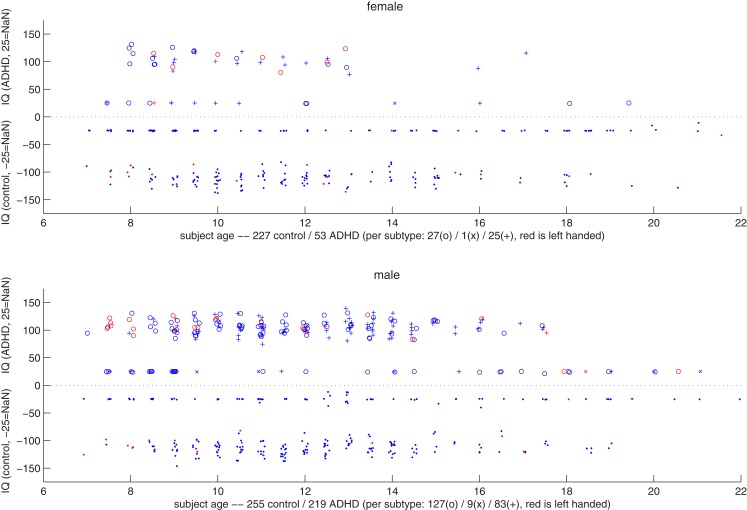
**Top: scatter plot representing female subjects from all sites, their DX status, age, combined IQ (average of the two available measures), and handedness**. The x-axis corresponds to the subject age. Along the y-axis, typically developing control subjects are plotted below the *y* = 0 line, and ADHD subjects above (i.e., |IQ| = IQ, with negative values simply denoting TDC subjects). The position along the y-axis corresponds to the average IQ measure, with subjects whose records did not include IQ plotted around *y* = − 25 and 25. The marker for each subject corresponds to diagnosis status (dot is control, o is ADHD-combined type, × is ADHD-hyperactive/impulsive, and + is ADHD-inattentive), and its color to handedness (red is left-handed). Coordinates are jittered slightly to improve visualization. Bottom: same as the plot above, but for male subjects.

Figure 2 shows a summary scatter plot of most of the non-imaging phenotypic information available for subjects, plotted separately for males and females across all performance sites. These plots summarize age, IQ, handedness, and diagnosis. By inspection it was clear that the ratio of control subjects to ADHD subjects was different between genders, and that within each gender that ratio was non-uniform across ages. Based on these determinations, we decided to stratify the dataset, treating each gender as a separate classification problem. The results reported below, unless otherwise noted, used this approach, and results represent the total performance levels across the gender-specific classifiers.

### Predicting ADHD from non-imaging phenotypic features

The non-imaging phenotypic feature set (age, gender, handedness, verbal and performance IQ, and binary site variables) provided substantial predictive power. Only 14 phenotypic features were available, and the results for predicting ADHD diagnosis using only these features (or subsets of these features) are described in Table 1. Performance according to the AUC measure is well above chance on the folds not used for training during cross-validation, even for 5 features, and reaches a level of AUC ≈ 0.81 (maximum possible value of 1.0 for a perfect binary classifier) using all available phenotype features. Performance is similar for the three different feature selection methods. The AUC drops considerably, however, on the separate test set (when trained on the complete training set), with maximum values of ∼0.72 for each feature selection method.

**Table 1:** **Non-imaging phenotype features only**.


**Cross-validation**	**5**	**10**	**All (14)**

2 Sample *t*-test	0.70	0.78	0.81
Nested CV	0.72	0.76	0.81
Recursive FE	0.73	0.80	0.81

**Test set**	**5**	**10**	**All (14)**

2 Sample *t*-test	0.71	0.71	0.72
Nested CV	0.70	0.70	0.72
Recursive FE	0.69	0.66	0.72

*Results summarizing ADHD prediction using non-imaging phenotypic features only. Entries indicate the area under the ROC curve (AUC) for classifiers built using different feature selection methods (rows) and different numbers of features (columns). *Top*: results on leave-out folds during cross-validation. *Bottom*: results on separate test set based on training across all examples in the training/cross-validation set*.

### Predicting ADHD from anatomical features

The anatomical feature set included cortical thicknesses at uniformly sampled locations in the spherical atlas space, average overall cortical thickness, and volumes of individual cortical and subcortical structures. Using these features alone, classifier performance is again substantially above chance and comparable to the baseline performance established by using the non-imaging phenotype features only. These results are summarized in Table 2. Maximum performance on the cross-validation folds is achieved using the full feature set (*N* = 5081 anatomical features), with AUC ≈ 0.77, slightly lower than the maximum of ∼0.81 achieved in cross-validation for the non-imaging phenotype features. Performance again drops somewhat to AUC ≈ 0.74 on the separate test set. However, it should be noted that this value indicates slightly *better* generalization of these classifiers to new test subjects than is observed for classifiers based on the non-imaging phenotypic features only (see Table 1).

**Table 2:** **Anatomical features only**.


**Cross-validation**	**10**	**20**	**50**	**100**	**200**	**400**	**800**	**1200**	**2000**	**3000**	**4000**	**All (5081)**

2 Sample *t*-test	0.64	0.67	0.63	0.58	0.62	0.69	0.71	0.72	0.74	0.76	0.77	0.77
Nested CV	0.62	0.64	0.65	0.63	0.62	0.64	0.68	0.70	0.75	0.77	0.76	0.77
Recursive FE	0.64	0.63	0.63	0.67	0.69	0.72	0.74	0.76	0.77	0.77	0.77	0.77

**Test set**	**10**	**20**	**50**	**100**	**200**	**400**	**800**	**1200**	**2000**	**3000**	**4000**	**All (5081)**

2 Sample *t*-test	0.74	0.76	0.73	0.69	0.61	0.61	0.68	0.70	0.70	0.72	0.74	0.74
Nested CV	0.67	0.70	0.74	0.67	0.59	0.70	0.71	0.70	0.71	0.71	0.73	0.74
Recursive FE	0.68	0.63	0.52	0.54	0.63	0.71	0.74	0.75	0.75	0.74	0.74	0.74

*Results summarizing ADHD prediction using anatomical features only. Entries indicate the area under the ROC curve (AUC) for classifiers built using different feature selection methods (rows) and different numbers of features (columns). *Top*: results on leave-out folds during cross-validation. *Bottom*: results on separate test set based on training across all examples in the training/cross-validation set*.

### Predicting ADHD from network features

As described above, three types of functional connectivity networks were constructed based on the filtered rs-fMRI time course data extracted from 116 brain regions in each subject. For each network (*Corr*, *SIC*, and *Kappa*; see *Methods*), the complete set of network features were computed and provided to feature selection and classification methods.

Table 3 summarizes the predictive power of the extracted network features alone for each of the three network construction methods. By comparison with Tables 1 and 2 it is clear that network features do not, on their own, achieve the same predictive power that either non-imaging phenotype features or anatomical features achieve.

**Table 3:** **Network features only**.


**CORR NETWORKS**													
**Cross-validation**	**10**	**20**	**50**	**100**	**200**	**400**	**800**	**1200**	**2000**	**3000**	**4000**	**6000**	**All (7150)**

2 Sample *t*-test	0.64	0.66	0.64	0.58	0.57	0.60	0.63	0.65	0.65	0.66	0.66	0.67	0.67
Nested CV	0.64	0.63	0.60	0.58	0.56	0.56	0.62	0.64	0.63	0.65	0.66	0.67	0.67
Recursive FE	0.55	0.57	0.54	0.59	0.63	0.66	0.65	0.65	0.67	0.67	0.67	0.67	0.67

**Test set**	**10**	**20**	**50**	**100**	**200**	**400**	**800**	**1200**	**2000**	**3000**	**4000**	**6000**	**All (7150)**

2 Sample *t*-test	0.72	0.68	0.67	0.60	0.53	0.53	0.57	0.62	0.66	0.69	0.70	0.67	0.67
Nested CV	0.64	0.64	0.68	0.65	0.59	0.50	0.60	0.62	0.65	0.70	0.71	0.68	0.67
Recursive FE	0.58	0.59	0.53	0.56	0.60	0.64	0.65	0.63	0.65	0.65	0.66	0.67	0.67

**SIC NETWORKS**													
**Cross-validation**	**10**	**20**	**50**	**100**	**200**	**400**	**800**	**1200**	**2000**	**3000**	**4000**	**6000**	**All (7345)**

2 Sample *t*-test	0.63	0.64	0.62	0.58	0.64	0.67	0.69	0.70	0.71	0.72	0.72	0.74	0.74
Nested CV	0.65	0.67	0.67	0.62	0.62	0.65	0.67	0.68	0.71	0.73	0.73	0.73	0.74
Recursive FE	0.65	0.64	0.64	0.70	0.71	0.72	0.74	0.74	0.73	0.73	0.74	0.74	0.74

**Test set**	**10**	**20**	**50**	**100**	**200**	**400**	**800**	**1200**	**2000**	**3000**	**4000**	**6000**	**All (7345)**

2 Sample *t*-test	0.64	0.72	0.66	0.65	0.60	0.58	0.62	0.63	0.65	0.67	0.70	0.71	0.71
Nested CV	0.49	0.58	0.62	0.64	0.57	0.58	0.66	0.68	0.70	0.69	0.70	0.70	0.71
Recursive FE	0.66	0.67	0.61	0.61	0.66	0.71	0.71	0.72	0.71	0.71	0.72	0.71	0.71

**KAPPA NETWORKS**													
**Cross-validation**	**10**	**20**	**50**	**100**	**200**	**400**	**800**	**1200**	**2000**	**3000**	**4000**	**6000**	**All (7344)**

2 Sample *t*-test	0.66	0.64	0.61	0.61	0.60	0.61	0.61	0.62	0.62	0.63	0.64	0.66	0.66
Nested CV	0.59	0.60	0.62	0.61	0.60	0.61	0.60	0.62	0.62	0.65	0.64	0.64	0.66
Recursive FE	0.62	0.61	0.60	0.62	0.64	0.64	0.65	0.65	0.65	0.66	0.66	0.66	0.66

**Test set**	**10**	**20**	**50**	**100**	**200**	**400**	**800**	**1200**	**2000**	**3000**	**4000**	**6000**	**All (7344)**

2 Sample *t*-test	0.63	0.63	0.63	0.60	0.57	0.56	0.56	0.54	0.56	0.59	0.59	0.61	0.61
Nested CV	0.60	0.60	0.56	0.56	0.59	0.59	0.55	0.57	0.59	0.60	0.60	0.61	0.61
Recursive FE	0.56	0.59	0.51	0.57	0.55	0.59	0.58	0.59	0.60	0.61	0.62	0.61	0.61

*Results summarizing ADHD prediction using network features only, as calculated from 3 different network construction methods. Entries indicate the area under the ROC curve (AUC) for classifiers built using different network construction methods (major groupings), feature selection methods (rows within each grouping), and different numbers of features (columns). Within each network construction method, *Top*: results on leave-out folds during cross-validation; *Bottom*: results on separate test set based on training across all examples in the training/cross-validation set*.

Features extracted from Sparse regularized Inverse Covariance (*SIC*) networks performed best overall, yielding a maximal AUC in cross-validation of ∼0.74 when using all network features, dropping to ∼0.71 when testing on the separate test set. Neither Correlation-based network features nor features derived from networks built from the Kappa statistic provided as much predictive power as the *SIC* networks, either in cross-validation or on the test set. Based on this result, only features derived from *SIC* networks were considered when combining feature sets as described below.

### Predicting ADHD from network features

**Figure 3:**
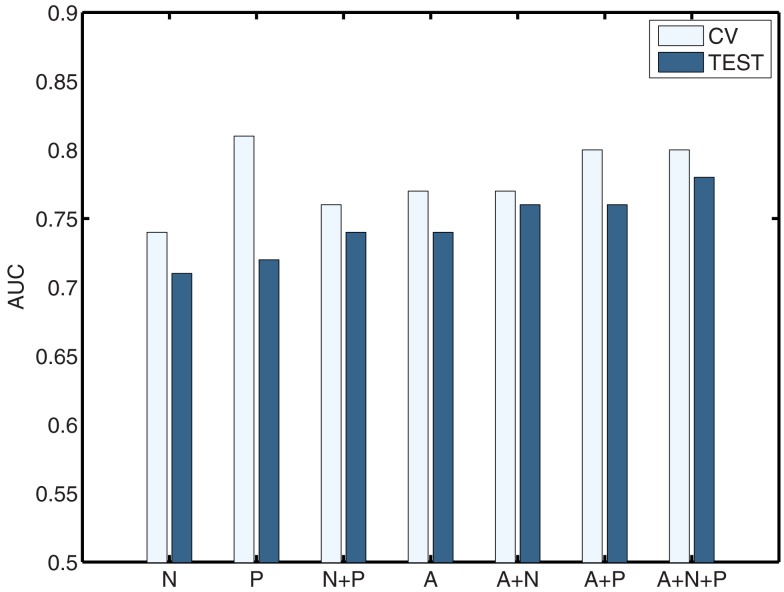
**Summary of best-performing classifiers using all features in the set or combination of sets below, sorted by performance on the test set (dark bars)**. Light bars indicate performance on non-training folds during cross-validation. A, Anatomical features; P, Non-imaging Phenotype features; and N, Network features.

The classification results obtained through using *all* of the available features from different combinations of feature sets are summarized in Figure 3, in which the x-axis is sorted by AUC on the *test set*. The best-performing classifier (all of which used stratification by gender, see *Methods*) used all features from all classes; in general combining feature types improved performance in cross-validation and particularly so on the test set. We note, however, that adding network features to the non-imaging phenotype features markedly *reduced* performance during cross-validation (0.81 vs. 0.76), but resulted in *improved* performance (0.74 vs. 0.72) on the separate test set when using all features from both classes. Indeed the difference between cross-validation performance and performance on the test set is largest when using the non-imaging phenotype features only. Tables 4–7 detail the results for the classifiers that used more than one feature set, for different numbers of features used, and for each of the three feature selection methods.

**Table 4:** **Combined non-imaging phenotype and anatomical features**.


**Cross-validation**	**10**	**20**	**50**	**100**	**200**	**400**	**800**	**1200**	**2000**	**3000**	**4000**	**All (5095)**

2 Sample *t*-test	0.69	0.70	0.67	0.63	0.66	0.74	0.74	0.76	0.78	0.79	0.80	0.80
Nested CV	0.76	0.79	0.75	0.68	0.64	0.70	0.74	0.76	0.79	0.80	0.80	0.80
Recursive FE	0.60	0.61	0.56	0.63	0.73	0.77	0.78	0.79	0.80	0.80	0.80	0.80

**Test set**	**10**	**20**	**50**	**100**	**200**	**400**	**800**	**1200**	**2000**	**3000**	**4000**	**All (5095)**

2 Sample *t*-test	0.69	0.66	0.65	0.63	0.66	0.69	0.71	0.70	0.73	0.74	0.75	0.76
Nested CV	0.74	0.74	0.70	0.66	0.65	0.69	0.72	0.71	0.73	0.76	0.75	0.76
Recursive FE	0.60	0.61	0.62	0.63	0.68	0.71	0.73	0.75	0.75	0.76	0.76	0.76

*Results summarizing ADHD prediction using non-imaging phenotypic features combined with anatomical features (but not network features). Entries indicate the area under the ROC curve (AUC) for classifiers built using different feature selection methods (rows) and different numbers of features (columns). *Top*: results on leave-out folds during cross-validation. *Bottom*: results on separate test set based on training across all examples in the training/cross-validation set*.

**Table 5:** **Combined non-imaging phenotype and network features**.


**Cross-validation**	**10**	**20**	**50**	**100**	**200**	**400**	**800**	**1200**	**2000**	**3000**	**4000**	**6000**	**All (7359)**

2 Sample *t*-test	0.68	0.69	0.66	0.60	0.66	0.70	0.71	0.72	0.73	0.75	0.75	0.76	0.76
Nested CV	0.77	0.80	0.72	0.67	0.68	0.69	0.69	0.70	0.73	0.75	0.75	0.75	0.76
Recursive FE	0.63	0.62	0.59	0.64	0.72	0.74	0.75	0.75	0.75	0.76	0.76	0.76	0.76

**Test set**	**10**	**20**	**50**	**100**	**200**	**400**	**800**	**1200**	**2000**	**3000**	**4000**	**6000**	**All (7359)**

2 Sample *t*-test	0.69	0.72	0.69	0.67	0.60	0.56	0.64	0.65	0.68	0.70	0.73	0.74	0.74
Nested CV	0.60	0.61	0.64	0.65	0.58	0.59	0.65	0.70	0.71	0.71	0.72	0.72	0.74
Recursive FE	0.67	0.67	0.61	0.63	0.72	0.73	0.72	0.74	0.76	0.75	0.75	0.74	0.74

*Results summarizing ADHD prediction using non-imaging phenotypic features combined with *SIC* network features (but not anatomical features). Entries indicate the area under the ROC curve (AUC) for classifiers built using different feature selection methods (rows) and different numbers of features (columns). *Top*: results on leave-out folds during cross-validation. *Bottom*: results on separate test set based on training across all examples in the training/cross-validation set*.

**Table 6:** **Combined anatomical and network features**.


**Cross-validation**	**10**	**20**	**50**	**100**	**200**	**400**	**800**	**1200**	**2000**	**3000**	**4000**	**6000**	**All (12,426)**

2 Sample *t*-test	0.63	0.64	0.61	0.60	0.62	0.66	0.71	0.72	0.75	0.75	0.76	0.77	0.77
Nested CV	0.62	0.64	0.65	0.63	0.62	0.64	0.68	0.70	0.75	0.77	0.76	0.77	0.77
Recursive FE	0.65	0.65	0.65	0.69	0.72	0.73	0.76	0.76	0.76	0.77	0.77	0.77	0.77

**Test set**	**10**	**20**	**50**	**100**	**200**	**400**	**800**	**1200**	**2000**	**3000**	**4000**	**6000**	**All (12,426)**

2 Sample *t*-test	0.72	0.74	0.72	0.66	0.66	0.70	0.67	0.71	0.70	0.70	0.70	0.73	0.76
Nested CV	0.49	0.63	0.62	0.64	0.57	0.69	0.68	0.69	0.70	0.72	0.74	0.73	0.76
Recursive FE	0.74	0.73	0.69	0.67	0.69	0.70	0.72	0.72	0.76	0.75	0.77	0.77	0.76

*Results summarizing ADHD prediction using anatomical features combined with *SIC* network features (but not non-imaging phenotypic features). Entries indicate the area under the ROC curve (AUC) for classifiers built using different feature selection methods (rows) and different numbers of features (columns). *Top*: results on leave-out folds during cross-validation. *Bottom*: results on separate test set based on training across all examples in the training/cross-validation set*.

**Table 7:** **Combination of all feature classes**.


**AUC RESULTS**													
**Cross-validation**	**10**	**20**	**50**	**100**	**200**	**400**	**800**	**1200**	**2000**	**3000**	**4000**	**6000**	**All (12,440)**

2 Sample *t*-test	0.65	0.66	0.64	0.62	0.65	0.69	0.75	0.75	0.77	0.78	0.79	0.79	0.80
Nested CV	0.77	0.79	0.75	0.68	0.64	0.70	0.74	0.76	0.79	0.80	0.80	0.80	0.80
Recursive FE	0.66	0.64	0.63	0.66	0.75	0.77	0.78	0.78	0.79	0.80	0.80	0.80	0.80

**Test set**	**10**	**20**	**50**	**100**	**200**	**400**	**800**	**1200**	**2000**	**3000**	**4000**	**6000**	**All (12,440)**

2 Sample *t*-test	0.72	0.74	0.76	0.70	0.66	0.73	0.70	0.73	0.71	0.72	0.72	0.76	0.78
Nested CV	0.60	0.66	0.64	0.63	0.62	0.69	0.67	0.69	0.71	0.74	0.75	0.76	0.78
Recursive FE	0.63	0.63	0.61	0.60	0.69	0.72	0.73	0.75	0.77	0.79	0.78	0.78	0.78

**ACCURACY RESULTS**													
**Cross-validation**	**10**	**20**	**50**	**100**	**200**	**400**	**800**	**1200**	**2000**	**3000**	**4000**	**6000**	**All (12,440)**

2 Sample *t*-test	0.68	0.65	0.63	0.63	0.64	0.67	0.69	0.69	0.74	0.72	0.72	0.73	0.74
Nested CV	0.74	0.74	0.70	0.66	0.65	0.69	0.72	0.71	0.73	0.76	0.75	0.74	0.74
Recursive FE	0.66	0.63	0.65	0.66	0.70	0.73	0.73	0.73	0.73	0.73	0.73	0.73	0.74

**Test set**	**10**	**20**	**50**	**100**	**200**	**400**	**800**	**1200**	**2000**	**3000**	**4000**	**6000**	**All (12,440)**

2 Sample *t*-test	0.69	0.67	0.69	0.65	0.63	0.66	0.63	0.66	0.62	0.61	0.60	0.63	0.67
Nested CV	0.61	0.62	0.60	0.60	0.58	0.65	0.63	0.64	0.64	0.67	0.70	0.65	0.67
Recursive FE	0.62	0.62	0.62	0.64	0.64	0.64	0.67	0.65	0.66	0.68	0.68	0.68	0.67

*Results summarizing ADHD prediction using combination of all feature types (non-imaging, anatomical, and network features extracted from *SIC* networks). Entries indicate the area under the ROC curve (AUC) for classifiers built using different feature selection methods (rows) and different numbers of features (columns). *Top*: results on leave-out folds during cross-validation. *Bottom*: results on separate test set based on training across all examples in the training/cross-validation set. The corresponding accuracy results are shown below*.

Combining all three feature sets yields the overall best prediction performance with an AUC ≈ 0.78 on the test set (∼0.80 on leave-out folds in cross-validation). For these results, which are depicted in Table 7, we have also included classifier *accuracy* results. As noted above, these values are based on one point on the ROC curve, which optimizes total percent correct for the binary diagnosis. We see that overall accuracy is above chance, but – particularly on the test set – not at a level that would allow, at this stage, a confident binary diagnosis for all example subjects.

**Figure 4:**
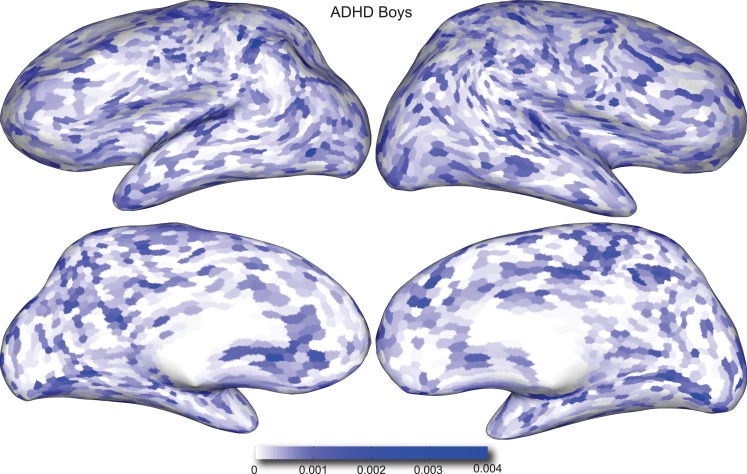
**Classification feature impact weights for cortical thickness features in the classification of boys with positive ADHD diagnosis**. Darker blue values indicate higher impact weights.

**Figure 5:**
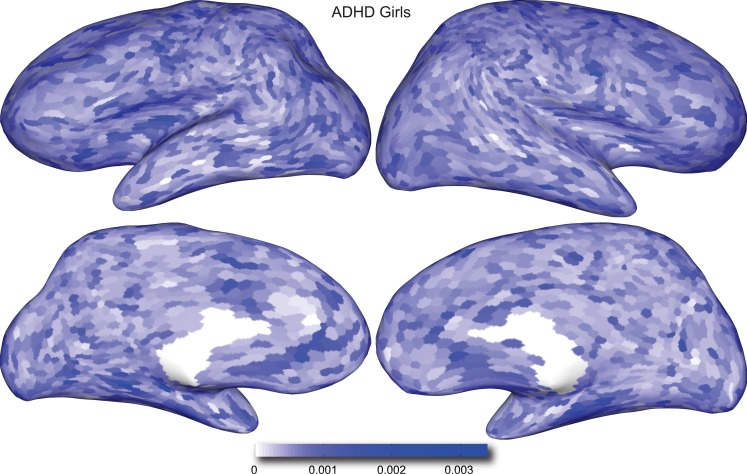
**Classification feature impact weights for cortical thickness features in the classification of girls with positive ADHD diagnosis**. Darker blue values indicate higher impact weights.

Figures 4 and 5 provide visualizations of the feature impact weights for *cortical thickness* features, which made up the majority of the anatomical feature set. These figures show the feature impacts for the ADHD-positive class; equivalent visualizations for the control class are available in Appendix. Despite the strong predictive power observed in non-imaging phenotype features alone, we observed that the combination of anatomical and network features *without* inclusion of the non-imaging phenotype features yielded the second best-performing classifier (tied with using anatomical and non-imaging phenotype features together) on new data, with an AUC ≈ 0.76 on the test set.

### Stratification

**Figure 6:**
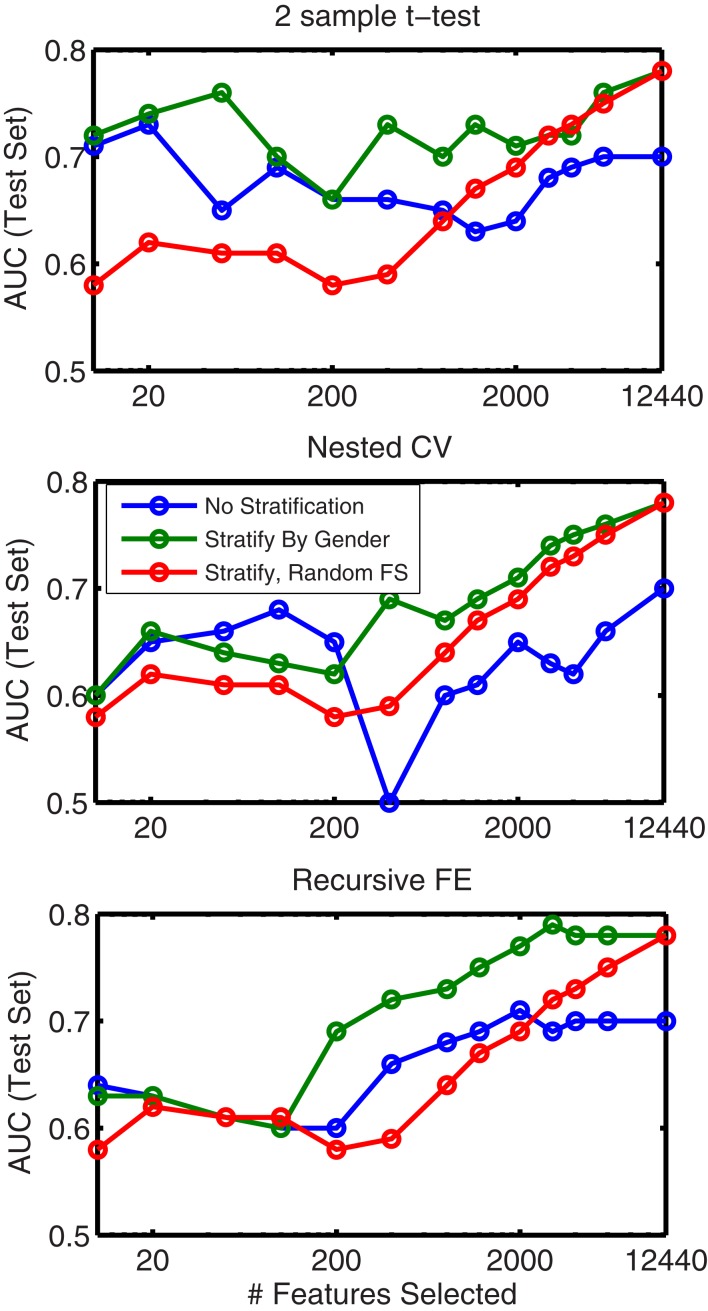
**Comparison of AUC performance on the test set as a function of number of features used for classifiers built from a combination of all feature classes, with (green line) and without (blue line) stratification by gender**. Each subplot represents results based on a different feature selection method. Also shown (red line, same in all plots) is the result obtained when features are ranked randomly (separately for each stratum); this result is averaged over classifiers built using 10 random feature rankings.

In order to evaluate the importance of stratifying by gender (which was used throughout our approach), classification using features from all feature sets (non-imaging, anatomical, and network) was repeated *without any stratification*. These results are shown in Table 8. Here we observed a dropoff in performance in cross-validation, but particularly a relatively large dropoff in AUC on the test set (AUC ∼ 0.70 using all features) in comparison with results that used separate gender-specific classifiers. Figure 6 demonstrates that the improvement due to stratification is systematic, plotting AUC on the test set for a range of numbers of selected features, and showing that stratification improves performance for the large majority of cases.

**Table 8:** **Combination of all feature classes without gender stratification**.


**Cross-validation**	**10**	**20**	**50**	**100**	**200**	**400**	**800**	**1200**	**2000**	**3000**	**4000**	**6000**	**All (12,440)**

2 Sample *t*-test	0.67	0.68	0.64	0.63	0.66	0.68	0.69	0.71	0.73	0.74	0.74	0.75	0.77
Nested CV	0.77	0.80	0.77	0.73	0.62	0.64	0.67	0.67	0.71	0.74	0.74	0.77	0.77
Recursive FE	0.63	0.64	0.65	0.67	0.68	0.71	0.73	0.73	0.75	0.76	0.76	0.77	0.77

**Test set**	**10**	**20**	**50**	**100**	**200**	**400**	**800**	**1200**	**2000**	**3000**	**4000**	**6000**	**All (12,440)**

2 Sample *t*-test	0.71	0.73	0.65	0.69	0.66	0.66	0.65	0.63	0.64	0.68	0.69	0.70	0.70
Nested CV	0.60	0.65	0.66	0.68	0.65	0.50	0.60	0.61	0.65	0.63	0.62	0.66	0.70
Recursive FE	0.64	0.63	0.61	0.60	0.60	0.66	0.68	0.69	0.71	0.69	0.70	0.70	0.70

*Results summarizing ADHD prediction using combination of all feature types (non-imaging, anatomical, and network features extracted from *SIC* networks) *without stratification by gender*. Entries indicate the area under the ROC curve (AUC) for classifiers built using different feature selection methods (rows) and different numbers of features (columns). *Top*: results on leave-out folds during cross-validation. *Bottom*: results on separate test set based on training across all examples in the training/cross-validation set*.

In addition Figure 6 plots the results obtained from *randomly* ranking features, then training classifiers (separately for boys and girls) using the top *N* features from the random list (in which rankings were held constant across folds). This plot is an average across 10 randomly generated rankings. The results show that our data-driven methods for selecting *N* features improve performance over simply choosing features at random (for all *N*), but interestingly, using gender-specific classifiers with *randomly selected features* outperforms “intelligent” feature selection without gender stratification when the number of features used is large.

Because stratifying by gender was quite powerful, we tested adding an additional level of stratification, by age. In this case, classifiers were constructed for three different age groups (the 0th–25th percentile, 25th–75th percentile, and 75th–100th percentile of age) within each gender, thus resulting in 6 total classifiers. The results of applying this method to the full set of features are given in Table 9. Cross-validation performance was qualitatively similar compared to stratification by gender alone, but performance on the test set was slightly improved, reaching AUC ≈ 0.80 when using all features (which was slightly better than the performance predicted in cross-validation – AUC ≈ 0.79).

**Table 9:** **Combination of all feature classes with stratification by gender and age**.


**Cross-validation**	**10**	**20**	**50**	**100**	**200**	**400**	**800**	**1200**	**2000**	**3000**	**4000**	**6000**	**All (12,440)**

2 Sample *t*-test	0.63	0.64	0.61	0.66	0.68	0.72	0.75	0.75	0.77	0.77	0.78	0.78	0.79
Nested CV	0.75	0.77	0.68	0.63	0.67	0.72	0.75	0.77	0.79	0.80	0.80	0.79	0.79
Recursive FE	0.62	0.62	0.67	0.73	0.75	0.76	0.77	0.78	0.78	0.78	0.78	0.79	0.79

**Test set**	**10**	**20**	**50**	**100**	**200**	**400**	**800**	**1200**	**2000**	**3000**	**4000**	**6000**	**All (12,440)**

2 Sample *t*-test	0.72	0.71	0.67	0.68	0.67	0.66	0.71	0.73	0.76	0.77	0.79	0.79	0.80
Nested CV	0.67	0.65	0.67	0.62	0.67	0.69	0.70	0.72	0.75	0.76	0.77	0.78	0.80
Recursive FE	0.59	0.55	0.64	0.64	0.72	0.76	0.78	0.80	0.80	0.80	0.80	0.80	0.80

*Results summarizing ADHD prediction using combination of all feature types (non-imaging, anatomical, and network features extracted from *SIC* networks) *with stratification by gender and age*. Entries indicate the area under the ROC curve (AUC) for classifiers built using different feature selection methods (rows) and different numbers of features (columns). *Top*: results on leave-out folds during cross-validation. *Bottom*: results on separate test set based on training across all examples in the training/cross-validation set*.

### Prediction results by subtype

**Figure 7:**
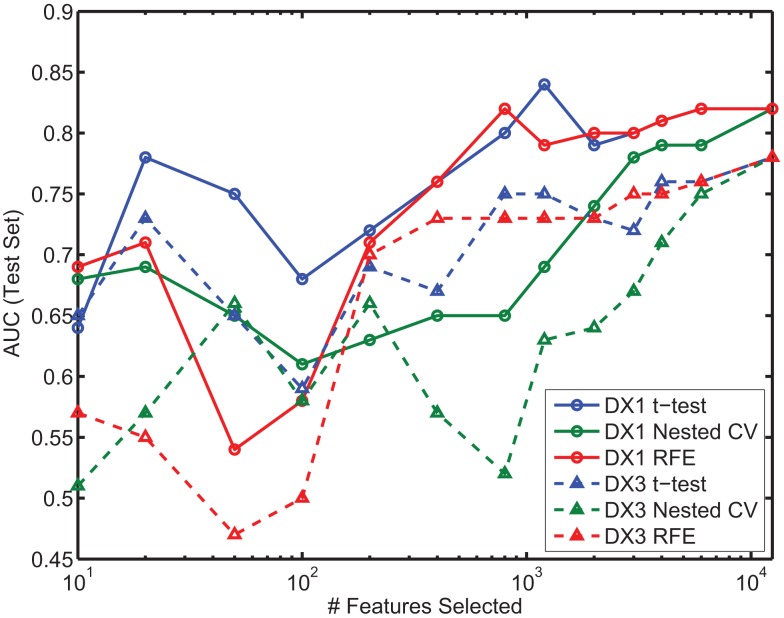
**Comparison of AUC performance on test set as a function of number of features used for the two predominant ADHD subtypes**. DX1 is the ADHD-Combined subtype; DX3 is the ADHD-Inattentive subtype.

In the present study we did not focus on predicting ADHD subtypes. However, most patients in the provided dataset were categorized as either ADHD-Combined Type (DX 1) or ADHD-Inattentive Type (DX 3), and we were interested in the question of whether one of these subtypes was easier to distinguish from controls than the other. We thus calculated performance of the classifier using all available features (e.g., as presented in Table 7), over two different datasets, each containing all the controls and patients from one of the subtypes. Figure 7 shows the results on the test set for each subtype, using each feature selection method, as a function of the number of features selected; each color is associated with one of the methods, with solid and dashed lines corresponding to ADHD-Combined and ADHD-Inattentive results, respectively. Regardless of the feature selection method used, AUC performance is almost always higher in the task of distinguishing ADHD-Combined from controls than it is for distinguishing ADHD-Inattentive from the same controls.

### Significance of classification results

The various sets of results are reported in the above tables without an explicit measure of statistical significance. There is no simple analytical model for the distribution of the AUC under the null hypothesis that the classifier is performing at chance, as there is for accuracy. One of the main goals of this paper is to contrast the results obtained using different feature types, and also the extent to which information is present across many features and, thus, we believe it is useful to report these results comprehensively. This poses a multiple comparisons problem, however, which is further complicated by the fact that the results obtained with each feature ranking method – a table row – are potentially correlated, since the top 20 features contain the top 10, the top 50 contain the top 20, and so on. Given that we do want to report results comprehensively, the canonical solution of using cross-validation within the training set to determine a single number of features to use is not desirable.

Perhaps the most elegant approach to test significance for an entire table of results would be to use permutation testing (Golland and Fischl, 2003). Unfortunately, given that each experimental run, using nested cross-validation for feature selection, is computationally expensive, it is impractical to run a sufficiently large number of permutations for each of the experimental conditions. Here we have opted to perform a smaller number (*N* = 100) of permutations of the results in Table 7 (which describes the best-performing classifiers based on using all feature types), and we report the mean AUC and SD of the estimate of the mean AUC in Table 10, as well as the analogous numbers for accuracy in Table 11. In each training set, the category labels used to select features and train a classifier were permuted, separately for each fold in the cross-validation case; in this manner, the balance of examples in each class was maintained, as was the stratification approach. It is important to note that we should expect permutation test accuracy results to be somewhat above 0.5 because there are unequal numbers of examples in each class.

**Table 10:** **Permutation tests of Table 7 results (AUC)**.


**Cross-validation**	**10**	**20**	**50**	**100**	**200**	**400**	**800**	**1200**	**2000**	**3000**	**4000**	**6000**	**All (12,440)**

**MEAN ACROSS 100 PERMUTATIONS**													
2 Sample *t*-test	0.581	0.575	0.557	0.536	0.525	0.542	0.560	0.572	0.582	0.589	0.594	0.598	0.602
Nested CV	0.593	0.583	0.561	0.542	0.525	0.543	0.558	0.564	0.574	0.583	0.585	0.589	0.602
Recursive FE	0.565	0.552	0.530	0.563	0.583	0.593	0.598	0.598	0.600	0.601	0.601	0.602	0.602
**SD OF MEAN ACROSS 100 PERMUTATIONS**													
2 Sample *t*-test	0.005	0.004	0.003	0.003	0.003	0.003	0.004	0.004	0.005	0.005	0.005	0.005	0.006
Nested CV	0.006	0.005	0.004	0.003	0.003	0.003	0.004	0.004	0.004	0.005	0.005	0.005	0.006
Recursive FE	0.004	0.004	0.003	0.004	0.005	0.005	0.005	0.005	0.005	0.006	0.006	0.006	0.006

**Test set**	**10**	**20**	**50**	**100**	**200**	**400**	**800**	**1200**	**2000**	**3000**	**4000**	**6000**	**All (12,440)**

**MEAN ACROSS 100 PERMUTATIONS**													
2 Sample *t*-test	0.566	0.580	0.565	0.549	0.527	0.529	0.544	0.554	0.562	0.569	0.579	0.586	0.600
Nested CV	0.563	0.553	0.549	0.534	0.522	0.529	0.543	0.554	0.564	0.577	0.587	0.592	0.600
Recursive FE	0.562	0.561	0.543	0.527	0.554	0.570	0.584	0.589	0.594	0.596	0.599	0.599	0.600
**SD OF MEAN ACROSS 100 PERMUTATIONS**													
2 Sample *t*-test	0.010	0.009	0.008	0.006	0.005	0.005	0.006	0.006	0.007	0.008	0.008	0.009	0.010
Nested CV	0.007	0.006	0.006	0.006	0.005	0.005	0.006	0.006	0.007	0.008	0.009	0.009	0.010
Recursive FE	0.008	0.008	0.007	0.006	0.007	0.008	0.009	0.009	0.009	0.010	0.010	0.010	0.010

*Results summarizing ADHD prediction using combination of all feature types (non-imaging, anatomical, and network features extracted from *SIC* networks) *with stratification by gender* in the permutation testing framework. Entries indicate the area under the ROC curve (AUC) for classifiers built using different feature selection methods (rows) and different numbers of features (columns). *Top*: results on leave-out folds during cross-validation. *Bottom*: results on separate test set based on training across all examples in the training/cross-validation set*.

**Table 11:** **Permutation tests of Table 7 results (accuracy)**.


**Cross-validation**	**10**	**20**	**50**	**100**	**200**	**400**	**800**	**1200**	**2000**	**3000**	**4000**	**6000**	**All (12,440)**

**MEAN ACROSS 100 PERMUTATIONS**													
2 Sample *t*-test	0.630	0.620	0.573	0.564	0.575	0.591	0.606	0.613	0.617	0.620	0.621	0.622	0.626
Nested CV	0.626	0.623	0.583	0.565	0.577	0.595	0.605	0.610	0.616	0.621	0.621	0.621	0.626
Recursive FE	0.592	0.593	0.609	0.615	0.618	0.620	0.625	0.625	0.625	0.624	0.626	0.626	0.626
**SD OF MEAN ACROSS 100 PERMUTATIONS**													
2 Sample *t*-test	0.002	0.001	0.002	0.002	0.002	0.002	0.002	0.002	0.002	0.002	0.002	0.002	0.002
Nested CV	0.002	0.003	0.002	0.002	0.002	0.002	0.002	0.002	0.001	0.002	0.002	0.002	0.002
Recursive FE	0.002	0.002	0.002	0.002	0.002	0.002	0.002	0.002	0.002	0.002	0.002	0.002	0.002

**Test set**	**10**	**20**	**50**	**100**	**200**	**400**	**800**	**1200**	**2000**	**3000**	**4000**	**6000**	**All (12,440)**

**MEAN ACROSS 100 PERMUTATIONS**													
2 Sample *t*-test	0.557	0.569	0.570	0.541	0.544	0.558	0.565	0.570	0.575	0.580	0.582	0.586	0.582
Nested CV	0.558	0.553	0.540	0.529	0.532	0.536	0.556	0.561	0.565	0.575	0.577	0.581	0.582
Recursive FE	0.557	0.555	0.562	0.568	0.575	0.584	0.583	0.582	0.579	0.580	0.581	0.582	0.582
**SD OF MEAN ACROSS 100 PERMUTATIONS**													
2 Sample *t*-test	0.002	0.003	0.004	0.004	0.004	0.004	0.003	0.003	0.003	0.003	0.003	0.003	0.003
Nested CV	0.003	0.003	0.003	0.004	0.004	0.003	0.004	0.004	0.003	0.004	0.003	0.003	0.003
Recursive FE	0.004	0.004	0.004	0.003	0.003	0.003	0.003	0.003	0.004	0.003	0.003	0.003	0.003

*Results summarizing ADHD prediction using combination of all feature types (non-imaging, anatomical, and network features extracted from *SIC* networks) *with stratification by gender* in the permutation testing framework. Entries indicate the accuracy for classifiers built using different feature selection methods (rows) and different numbers of features (columns). *Top*: results on leave-out folds during cross-validation. *Bottom*: results on separate test set based on training across all examples in the training/cross-validation set*.

These results, in comparison with Table 7, demonstrate that our best-performing classifiers are performing well above empirically defined chance levels. For example, the mean AUC and accuracy (ACC) scores for classifiers using all 12,440 features under class label permutations is AUC = 0.602 ± 0.006 and ACC = 0.626 ± 0.002 under cross-validation, compared to an observed values of AUC = 0.80 and ACC = 0.74 for the classifier trained with correct class labels. This large difference holds up in the test set as well, where the empirical chance values are AUC = 0.60 ± 0.01 and ACC = 0.582 ± 0.003 and the correctly trained classifier values are AUC = 0.74 and ACC = 0.67.

### Prediction results by site

We examined the performance of the *best classifier* (with stratification by gender only, based on using all features from all feature classes) as a function of the *site of performance* of the imaging session. Here we used accuracy as the outcome measure, and separated the corresponding errors into false positives and false negatives. These results represent a single point from an ROC curve. Our goal was to show the variability in performance across sites and the types of errors made within each site. These results are depicted in Table 12 for both cross-validation and on the separate test set.

**Table 12:** **Results of best overall accuracy predictor by site for predictions based on the full set of combined features**.


**Site**	**FP error**	**FN error**	**Accuracy**	**# Subjects**

**CROSS-VALIDATION**				
Peking University	0.07	0.18	0.75	189
Kennedy Krieger Institute	0.10	0.22	0.69	83
NeuroIMAGE Sample	0.10	0.27	0.62	48
New York University Child Study Center	0.14	0.18	0.68	213
Oregon Health and Science University	0.07	0.37	0.56	73
University of Pittsburgh	0.03	0.00	0.97	89
Washington University in St. Louis	0.02	0.00	0.98	59
**TEST SET**				
Peking University	0.06	0.32	0.62	50
Kennedy Krieger Institute	0.00	0.18	0.82	11
NeuroIMAGE Sample	0.00	0.35	0.65	23
New York University Child Study Center	0.10	0.22	0.68	41
Oregon Health and Science University	0.09	0.18	0.74	34
University of Pittsburgh	0.11	0.44	0.44	9
Washington University in St. Louis	N/A	N/A	N/A	0

## Discussion

The above results demonstrate that it is possible to predict the diagnosis of Attention-Deficit/Hyperactivity Disorder within the set of available subjects to a level of certainty that is well above what would be expected by chance (naively AUC ≈ 0.5, but empirically evaluated under certain conditions as shown in Table 10) using many combinations of non-imaging phenotype features and/or MRI-based anatomical or resting-state functional network features. We took a systematic approach to testing the predictive power of each of the three main feature sets on their own (Tables 1– 3) and then in combination (Tables 4– 7). The results using all features from those sets are summarized in Figure 3. In general, the small set of non-imaging phenotypic features were able to provide a large fraction of the available predictive power. However, imaging features, when used in large numbers, were able to boost performance by improving generalization to a separate test set of subjects. Such anatomical and rs-fMRI network features were also able to provide very strong predictions on their own, while using only gender as a non-imaging basis for stratifying the classification problem.

In this paper we measured prediction performance using the area under the ROC curve (AUC) rather than the simpler measure of *accuracy* (i.e., rate of correct classification) because accuracy can be a misleading measure under certain circumstances. In particular, when classes are unbalanced, as is often the case in clinical diagnostics (i.e., there will be many more control cases than disorder cases), high accuracy can be achieved simply by biasing the classifier heavily toward the control class. ROC curves thus are an important approach for evaluating clinical tests (Zweig and Campbell, 1993) because they plot the true positive rate against the false positive rate, establishing the area under the ROC curve as a measure of discrimination between classes across the full operating range of the classifier. By comparison, the use of accuracy as the measure of performance in this two-class problem forces evaluation of the classifier at a particular threshold, above which one call is made, and below which the other is made. In a real-world clinical setting it may be useful to *tune* the classifier to highlight either *sensitivity* to detect true cases of ADHD at the expense of a higher false positive rate, or to increase *specificity* to reduce false positive rate at the expense of a higher false negative rate. Thus AUC provides a more general measure that is applicable in the current context. Still, because accuracy is a well-recognized and commonly reported performance measure, we have included such results in the case of classifiers built from all feature types (Table 7).

### Stratification by gender

Based on current knowledge of ADHD, there are compelling reasons to believe that diagnostic aids might perform better if tailored to boys separately from girls, and this is reflected in our results. First, ADHD is diagnosed at a significantly higher rate in boys than in girls (Polanczyk et al., 2007), and thus the overall probability of an ADHD-positive diagnosis – averaged across other factors – should be different between genders. Still, it is possible that the same *features* (and weightings of those features) are important in diagnosing ADHD in both genders, and that those features are simply present in different proportions between genders. If this were the case, then a single classifier trained using those features should perform equally well compared with gender-specific classifiers, but this was not the case. Tables 7 and 8 show results using all feature sets with and without stratification by gender. The performance is notably reduced, particularly in the test set, using a single classifier. Figure 6 demonstrates that this stratification effect is quite consistent for different numbers of selected features.

These results are consistent with the literature suggesting neurobehavioral gender differences in individuals with ADHD that go beyond mere prevalence (Gaub and Carlson, 1997; Castellanos et al., 2001; Newcorn et al., 2001; Gershon and Gershon, 2002; Mahone and Wodka, 2008). In the present study, the gender-specific classifiers that combined all non-imaging phenotypic, anatomical, and network features weighted the available imaging features substantially differently. For the classifiers using the entire feature set, the Pearson correlation coefficient between the feature weight vector for boys and the feature weight vector for girls was *r* = 0.0757. Although small, this represented a significant correlation in this high-dimensional space (*p* < 1 × 10^−13^). Qualitative comparison of the feature impact weights points to many differences (see Supplementary Material for a full list of features and their impact weights for each class, with local attributes linked to anatomy through the AAL nomenclature (Tzourio-Mazoyer et al., 2002) for network features or the nomenclature of the (Desikan et al., 2006; FreeSurfer parcellation system). To provide one such example, highly impactful features for boys with ADHD include a series of network edge weights connecting cortical and striatal structures (5 of the top 50 ranked features) whereas none of these features appear in the top 50 for girls with ADHD.

An additional level of stratification was tested for the classifiers that used all feature sets, in this case including sub-classifiers for age groups. These groups were defined by percentiles (0–25, 25–75, and 75–100) within the age range spanned by the training set within each gender group. While this set of stratified classifiers yielded quantitatively the *best* overall performance (AUC ∼ 0.79 in both cross-validation and on the separate test set), we focused more here on gender-only stratification because further splitting of the dataset results in relatively small sample-size partitions, and we lack overall confidence that such results will continue to hold over new, larger datasets. Still, stratification by age is a sensible approach given sufficient data given the considerable developmental changes that are occurring over the window of ages considered here, including the maturation of resting-state functional connectivity networks in typically developing children (Fair et al., 2008).

### Phenotypic features

Non-imaging phenotype features included gender, age, handedness (which was coded as an integer – left/right/ambidextrous – for most sites, but as a decimal based on the Edinburgh Handedness Inventory for patients scanned at NYU), and performance and verbal IQ (which were missing for some patients and were thus replaced with the population mean value when training). In addition, binary features were created to indicate the absence of an IQ score as well as eight features to indicate at which of the eight possible sites the scan data were obtained.

These features had substantial predictive power and provided a high baseline for image feature-based classifiers to surpass. The maximal performance using these non-imaging attributes only was achieved using all 14 available phenotypic features (both in cross-validation and on the separate test set), but performance dropped off substantially from the 0.81 AUC obtained in cross-validation to 0.72 on the separate test set. This reduction in performance may indicate that the distributions of these phenotypic features in the test set did not sufficiently mirror those found in the training set. Overall, the population of patients and typically developing controls for whom data were made available were not necessarily representative of the population at large. For example, ∼36% of training samples had an ADHD-positive diagnosis, and the male-to-female ratio within that sample was not reflective of overall diagnosis rates. While the dataset used here was *large* relative to many single studies of ADHD, the numbers are still potentially too small given the overall population variance in measures like IQ to enable completely effective prediction based on this small set of features.

### Imaging features

A large number of features were calculated based on each patient’s imaging data. These measures were subdivided into *anatomical features*, which were processed in one stream, and *network features*, which were processed in another (see Figure 1).

#### Anatomical features

The anatomical feature set was dominated by estimates of cortical thickness at 2,562 locations per hemisphere, a small fraction of which were excluded because they represented non-cortical areas and were set to be constant across subjects. The cortical thickness locations used corresponded to the vertices comprising an icosahedral approximation to a sphere in the FreeSurfer spherical atlas space that was used for anatomical inter-subject registration. This yielded a relatively low-resolution resampling of each subject’s thickness data, which was performed in order to reduce dimensionality to accommodate machine learning techniques, to improve estimates of local thickness by effectively averaging over small neighborhoods, and to allow for the expected small registration errors without destroying subject-to-subject feature correspondences.

Cortical thickness has previously been shown to be a relevant biomarker in ADHD (e.g., Shaw et al., 2006; Makris et al., 2007). Many cortical thickness values were assigned relatively large feature weights (see Supplementary Material) in the classifiers built using all features from all feature sets; the *feature impact weights* (see *Methods*) for ADHD-positive diagnosis for boys (Figure 4) and girls (Figure 5) were rendered on inflated cortical surface models for purposes of visualization. While some of the particular nodes assigned high impact are in areas previously shown to have differences in cortical thickness in subjects with ADHD, in general the thickness impacts are distributed across cortex and not focused in one or a few brain areas, and thus are difficult to interpret succinctly in a biological context. Within the group of cortical thickness features with impacts at least 2 SD above the median (i.e., those that were particularly important in diagnosis), ∼53% were in the right hemisphere in boys whereas only ∼42% were in the right hemisphere in girls.

The caudate nucleus has been considered a structure of interest in ADHD due to reports of volume differences in patients (Castellanos et al., 1994). In our results, left and right caudate volume (normalized by intracranial volume) each were assigned high impact for ADHD diagnosis in girls (237th and 345th most highly impactful features, respectively, each of which represented an impact that was more than 2 SD above the median feature impact weight for that class), but not in boys. We did not directly include measures of *asymmetry* (though in principle this could be computed implicitly by the existing classifier), which may be particularly useful, as it has been claimed that the degree of caudate asymmetry may reflect severity of ADHD symptoms in children (Schrimsher et al., 2002). The only other structure volume feature to receive similarly high impact was the volume of the right inferior lateral ventricle in boys, which ranked 450th in impact for the control class and 506th in impact for the ADHD-positive class. This feature did not rank highly for girls.

#### Network features

Our corpus of network-based features included a variety of standard features found commonly in functional connectivity studies as well as a series of features drawn from other scientific domains or developed for this study. These included measures of small-worldness and scale-free properties, node-level connectivity or other measures of “importance” (using single path and parallel path measures), network separability, network sparsity, and network motifs. This broad base of network feature types allowed us to systematically examine the power of features by exploring which were selected and/or had large impact on the classification problem. Plots showing the mean and SD feature impacts for each gender, calculated across *categories* of features (including both network and anatomical features) based on the classifier built using all features of all types is available in the Appendix.

We calculated the set of network features after building rs-fMRI functional connectivity networks using three different methods: correlation (*Corr*), the inverse of the covariance matrix under L1-norm regularization to promote sparsity (*SIC*), and Patel’s Kappa measure (*Kappa*). These network construction methods were chosen largely based on the results of Smith et al. (2011), as they were among the top performing methods in simulation studies for correctly inferring underlying connectivity (without direction). In the present results (see Table 3) we observed a rather clear advantage for using network measures derived from the *SIC* networks relative to the other two methods; this advantage was predicted by better overall results throughout cross-validation (particularly for large numbers of features), as well as better results on the separate test set (AUC ≈ 0.71 vs. 0.67 for *Corr* networks and 0.61 for *Kappa* networks, when using all network features).

Many network features were among those that had high impact weights for classifiers that used all features (with stratification by gender). Because each edge weight (of 6670 possible edges in the AAL network) was used as a feature, these tended to dominate lists of highly ranked features, but other derived network properties were also impactful. For convenience, we again examined impact weights that were greater than 2 SD from the mean for that class. While individual features are difficult to interpret, a broad picture emerges pointing to a role for functional connectivity attributes involving multiple cerebellar areas and primarily frontal cortical areas, especially in the right hemisphere.

Beyond individual edge weights, weighted node degrees (which are a measure of the overall estimated functional connectivity of that brain area with all other regions), and node betweenness (which can be thought of as a node’s *importance* if the network represents flow of information or activity) of frontal cortical and cerebellar nodes often had high impact. The second most impactful feature for diagnosis of girls with ADHD was the degree of the left median cingulate cortex, while the impact of betweenness of that same node ranked fifth overall. Highly impactful node degree features were found within the cerebellum in both girls (right lobules III, VI, VIIB, and Crus II; left lobule III; and vermal lobules I, II, and X) and boys (right cerebellum lobules VI and IX, vermal lobule III), as were node betweenness features (left lobule III; right lobule VI and Crus II, vermal lobules I, II, and X in girls; right lobule VI, vermal lobule III in boys). Yu-Feng et al. (2007) found that children with ADHD had reduced amplitude of low-frequency fluctuations (in the approximate frequency band analyzed here) bilaterally in the cerebellar cortex and vermis, which might drive reductions in weights of the functional connections with cerebellar regions in our networks, resulting in differences in subtle group-level features like node degree or betweenness.

Additional non-standard network features that had relatively high impact included central point dominance measures (e.g., in the left middle temporal pole, left middle and superior frontal cortex, and left superior occipital cortex in boys with ADHD), eccentricity measured by effective resistance (of the left precentral gyrus in girls with ADHD) and a variety of network sparsity features. In particular, sparsity measures based on lexicographic, Dulmage–Mendelsohn, and approximate minimum degree orderings (without applying an edge threshold) in girls with ADHD (and to a lesser extent boys with and without ADHD) were associated with relatively large feature impacts.

Overall, performance for classifiers based on network features alone was somewhat lower than performance using non-imaging phenotypic features or anatomical features. This seems to indicate that the individually derived features may be somewhat noisy, and perhaps an even larger sample size will be required to develop more robust network feature-based classifiers. It is possible that other derived features could hold substantially more predictive power than those used here, including, for example, additional measures of the degree of modularity or goodness or partitioning (e.g., Rubinov and Sporns, 2011), but the set of attributes we deployed was quite large and heterogeneous, for example including many features at both the local (node) level and the global (network) level.

Another important consideration for network-level features, beyond the network construction method we explored here, is the definition of the brain regions that comprise nodes. In the current project we defined each node as an anatomical region-of-interest using the AAL volumetric brain atlas (Tzourio-Mazoyer et al., 2002), which is a macro-anatomical parcellation of the MNI-space single-subject brain. Each ROI is then represented by a mean time course (after rs-fMRI preprocessing), which is assumed to be representative of regional activity. Smith et al. (2011) demonstrated, however, that improperly defined regions-of-interest can substantially degrade inference of connections (in any network construction method). Thus one avenue of further study to improve predictive power is to attempt to define regions-of-interest (and therefore rs-fMRI networks) in a more data-driven manner, perhaps by first clustering voxels into ROIs based on similarity of response.

The construction of network features depended on a set of pre-processing steps that adjusted the raw BOLD time series to examine low-frequency fluctuations, while attempting to account for a variety of nuisance variables. We used the Athena pipeline scripts to carry out a set of relatively standard pre-processing steps including regression of the mean time courses from white matter and CSF as well as of six time-varying parameters, which estimate translations and rotations due to subject head motion. It is important to note, however, that multiple studies (Power et al., 2012; Van Dijk et al., 2012) have now demonstrated that even including these parameters in the regression model may be insufficient to completely remove the effects of motion on estimates of functional connectivity. Such motion effects result in colored noise, giving rise to distance-dependent changes in correlation strengths, typically yielding an overestimate of local connectivity and an underestimate of long-range connectivity. While the data from Van Dijk et al. (2012) showed that head motion may not account for a large portion of the variance within a subject group, it may still be possible for *between-group* differences to be driven primarily or perhaps even entirely by differences in group motion profiles. Given the nature of the groups studied here – children, who are known to be prone to movement, including those with a clinical condition – results from network features should be interpreted with caution as they may include effects that are driven by head motion. A more thorough “scrubbing” procedure (Power et al., 2012) might be beneficial to remove high motion data frames that potentially contribute rapid, large BOLD changes and bias correlations. Still, it does not appear that regression of motion parameters *causes* artifactual estimates of functional connectivity, but instead may help to alleviate artifacts, at least partially (Power et al., 2012). How to estimate the extent of the impact of head motion, and how to completely remove such effects from group functional connectivity studies remain unresolved research questions, which will be important to carefully address as large data-driven studies become more prevalent.

### Combining feature sets

Figure 3 demonstrates classifier performance (in both cross-validation and on the separate test set) for classifiers that were built using *all available features* for different combinations of feature sets (defined as non-imaging phenotype, anatomical, and network sets). The best overall performance in cross-validation, which should generally be predictive of performance on the separate test set, occurred for the classifier built from non-imaging phenotype features only. This result was surprising (and somewhat discouraging for efforts to base diagnosis on biological measurables from imaging data), but did not robustly generalize when results were assessed on the separate test set. In particular, generalization of the non-imaging features seemed relatively weak, whereas adding additional features from the imaging sets (in sufficient numbers) appeared to improve the classifiers’ ability to generalize to new patient data. Adding only a small number of imaging features to the phenotype set actually *degraded* performance in almost all cases, but as features continued to be added, there was a slow, systematic increase in performance, generally reaching maximum when using *all* available features.

As seen in Figure 3, the best overall performance on the *test set* came using an SVM that weighted all 12,000+ features across the three feature sets. It is somewhat unusual that such a large feature set (relative to the number of training examples/subjects) would provide optimal performance. We see this as an indication of the diffuse nature of ADHD. Our results make it clear that there is substantial predictive power in the set of features derived from either anatomical scans, resting-state fMRI networks built from sparse inverse covariance estimates, or combinations of the two, but it also appears that, in general, no small set of these features can provide better predictions than the non-imaging phenotypic features on their own. Instead, predictions are honed and made more robust to new data by a combination of many features, each with small overall contributions to the final class prediction.

Upon initial review, these results may bring into question the relative merits – particularly from a cost/benefit point of view – of using neuroimaging-derived characteristics for the clinical diagnosis of ADHD, and perhaps more generally for diagnosis of neuropsychiatric disorders. While our results indicate an improvement in AUC of less than ∼10% in new test subjects when incorporating imaging features (both anatomical and network features) above and beyond the non-imaging feature set, we feel that this is an important improvement. Because ADHD diagnosis is controversial (Wolraich, 1999), any added ability to point to objective, biological measures is of high potential significance. One major objective for algorithmic diagnosis of behaviorally defined disorders is to find biomarkers that enable diagnosis in a manner that is indistinguishable from a group of physicians. In this dataset we only have one diagnosis, which was presumably given by one physician or a small group of physicians, and thus we cannot relate our results to the variability that might exist in a set of blind diagnoses from a group of physicians. Thus while we do not feel that the current measures are ready for the clinic, the appearance of modest but highly significant improvements of diagnosis prediction based on imaging features gives hope for such methods serving as a diagnostic aid in the future.

### Importance of large datasets

The ADHD-200 dataset has provided a new opportunity to apply exploratory data analysis, data mining, and machine learning tools to the incredibly challenging problem of neuropsychiatric disorder diagnosis. While there is great value in meta-analyses across sets of previously conducted studies, computational scientists place great value in the ability to apply the same methods directly to commonly coded datasets made publicly available in a common format. To achieve results that would directly impact ADHD diagnosis, it may be important to collect data from even more subjects.

We observed substantial differences in classifier performance across scan sites (Table 12); however, data were insufficient to properly train classifiers for each site, and we interpret these results cautiously due to relatively small numbers of examples. The variability in classifier performance by site may arise from at least two sources. First, it may reflect aspects of the patient populations that systematically differed by site. Furthermore, different sites used somewhat different acquisition protocols; standardizing these protocols and/or developing additional algorithmic methods to account for such differences may improve overall performance and reduce classifier differences across sites. The types of data assembled here would be well complemented with a variety of other measures, possibly including task-driven fMRI in, for example, set shifting or cognitive/inhibitory control tasks that highlight difficult patterns of behavior for subjects with ADHD. Further, ADHD has a significant genetic component (Faraone et al., 2005), and combining genotype or gene expression information with possible endophenotypes from brain imaging (i.e., imaging genetics; Durston et al., 2009) is a promising avenue that will also require collection of both brain imaging data using standardized protocols as well as genetic materials from a large number of patients and matched controls.

## Conclusion

The overall framework presented here, which combines different feature sets, each processed in distinct software streams, provides a flexible and extensible means for studying diagnostic measures in large clinical datasets. While it was somewhat surprising that classifiers, in general, achieved best performance using *all* available features, this points to the distributed nature of pathology in complex neuropsychiatric disorders, while establishing the need to combine many diverse attributes for best outcomes. The general approach and set of features described here will be useful in future examinations of nervous system disorders and also potentially for predictions of various brain states based on functional connectivity networks in task-active fMRI.

## Conflict of Interest Statement

The authors declare that the research was conducted in the absence of any commercial or financial relationships that could be construed as a potential conflict of interest.

## Author Contributions

Jason W. Bohland and Leo Grady conceived the study and overall approach. Sara Saperstein, Leo Grady, and Jason W. Bohland conducted the initial data analyses. Leo Grady provided algorithms for network measures. Francisco Pereira conducted additional analyses and developed and greatly enhanced the machine learning framework. Jérémy Rapin provided advice on network construction methods. Jason W. Bohland, Leo Grady, Francisco Pereira, and Sara Saperstein wrote the manuscript.

## Supplementary Material

The Supplementary Material for this article can be found online at http://www.frontiersin.org/Systems_Neuroscience/10.3389/fnsys.2012.00078/abstract

## Figures and Tables

**Figure A1:**
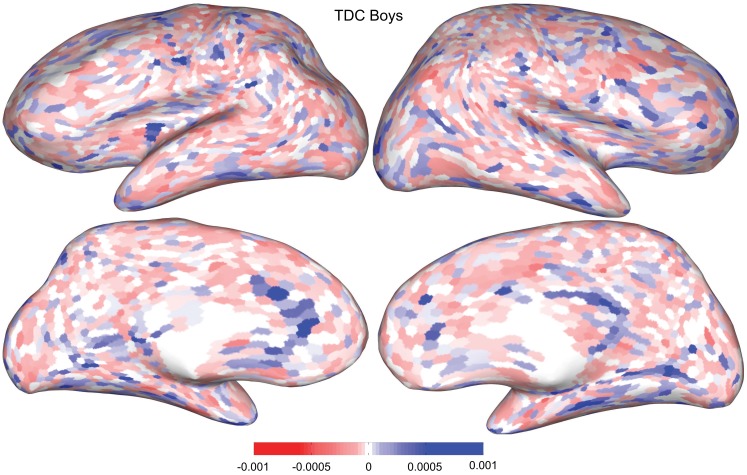
**Classification feature impact weights for cortical thickness features in the classification of boys as typically developing controls**.

**Figure A2:**
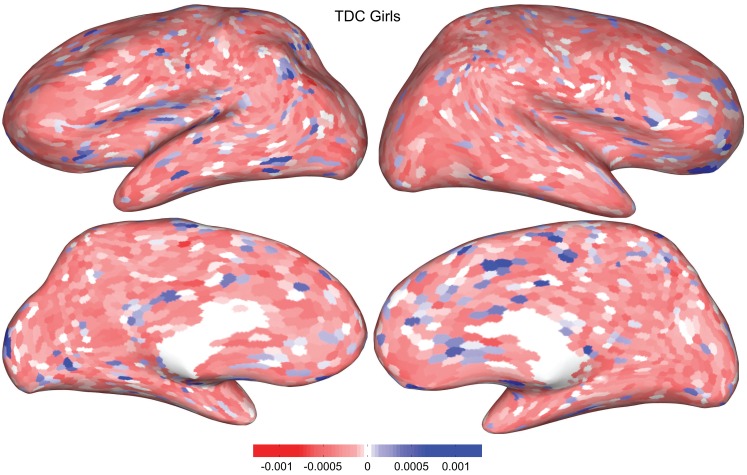
**Classification feature impact weights for cortical thickness features in the classification of girls as typically developing controls**.

**Figure A3:**
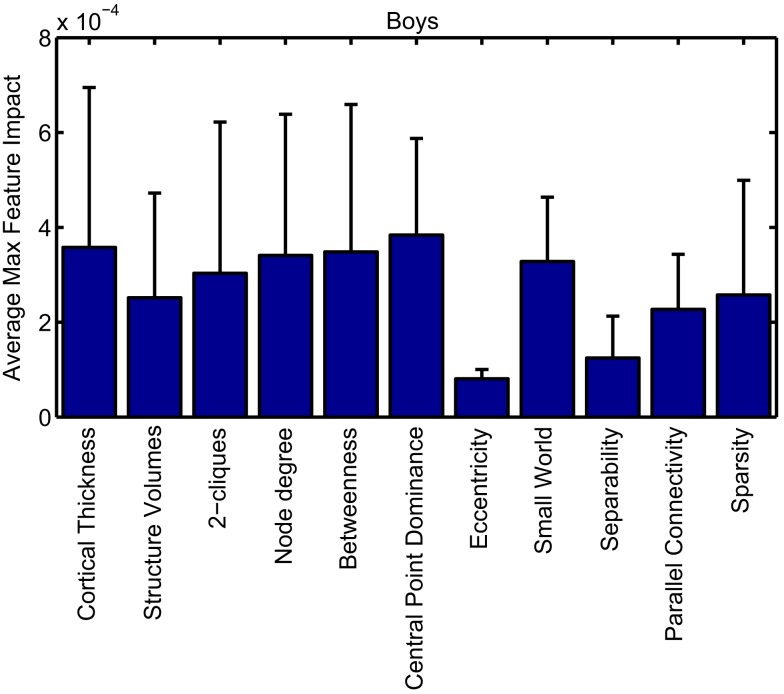
**Imaging feature impacts by category for boys**. Bar heights represent the average (across features) of the higher value (ADHD-class or TDC-class) feature impact weight for each feature of a certain category for the male-specific classifier (using all features). Error bars are SD of those values across the features in the category.

**Figure A4:**
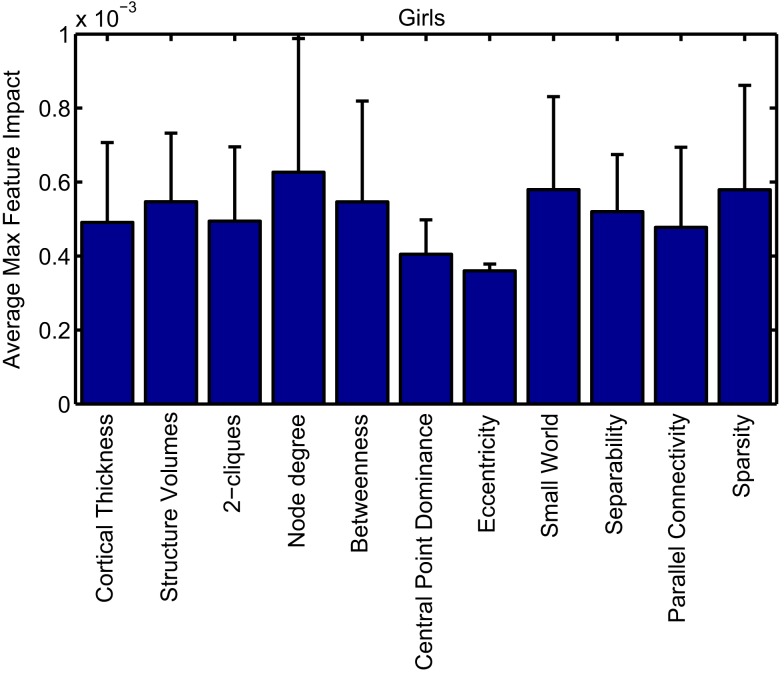
**Imaging feature impacts by category for girls**. Bar heights represent the average (across features) of the higher value (ADHD-class or TDC-class) feature impact weight for each feature of a certain category for the female-specific classifier (using all features). Error bars are SD of those values across the features in the category.

## Footnotes

^1^http://www.cdc.gov/ncbddd/adhd/data.html

^2^http://fcon_1000.projects.nitrc.org/indi/adhd200/

^3^The individual parameters for each site are available at the competition web site: http://fcon_1000.projects.nitrc.org/indi/adhd200/.

^4^http://surfer.nmr.mgh.harvard.edu

^5^http://neurobureau.projects.nitrc.org/ADHD200/Data.html

^6^For details of the Athena pipeline see http://www.nitrc.org/plugins/mwiki/index.php/neurobureau:AthenaPipeline

## Appendix

### Network measures

This appendix provides a brief introduction to a subset of the network measures used in the accompanying manuscript. These and other network measures are described in Grady and Polimeni (2010) *Discrete Calculus: Applied Analysis on Graphs for Computational Science*, Springer.

The *degree* of a node *i* in our weighted networks is calculated as:

$$\text{Degree}\left(v_{i}\right)=\underset{v_{j}}{\sum }\;w\left(e_{ij}\right)$$

where *w*(*e_ij_*) is the distance weight of edge *e_ij_* connecting nodes *i* and *j*.

The distance between any two nodes in the network may be calculated as the optimal, or shortest weighted path between those nodes:

$$D\left(v_{i},v_{j}\right)=\underset{\Pi_{i,j}}{\min}\underset{e_{ij}\in \Pi_{i,j}}{\sum }\;w\left(e_{ij}\right)$$

The *average path length* is then simply:

$$\frac{1}{N\left(N-1\right)}\underset{v_{i}}{\sum }\;\underset{v_{j}\neq v_{i}}{\sum }\;D\left(v_{i},v_{j}\right)$$

where *N* is the number of nodes in the network.

The *clustering coefficient* of a node counts the number of pairs of its neighbors (nodes to which it is connected) that are also connected. It is given by:

$$\text{CC}\left(v_{i}\right)=\frac{\#\text{of closed pairs of neighbors of}\;v_{i}}{\text{Total }\#\text{ of pairs of neighbors of}v_{i}}$$

and the average clustering coefficient is then simply:

$$\text{CC}\left(\text{G}\right)=\frac{1}{N}\underset{v_{i}}{\sum }\;\text{CC}\left(v_{i}\right)$$

The *eccentricity* of a node is the maximum distance between that node and any other node in the network:

$$\text{Eccentricity}\left(v_{i}\right)=\underset{v_{j}}{\max}D\left(v_{i},v_{j}\right)$$

The graph *diameter* is defined as:

$$\text{Diameter}\left(G\right)=\underset{v_{i}}{\max}\text{Eccentricity}\left(v_{i}\right)$$

and the graph *radius* is defined as:

$$\text{Radius}\left(G\right)=\underset{v_{j}}{\min}\text{Eccentricity}\left(v_{i}\right)$$

The node *betweenness* is a measure of the centrality or importance of a node, and is defined as:

$$\text{Betweenness}\left(v_{i}\right)=\underset{\begin{array}{c} v_{j},v_{k}\\ v_{i}\neq v_{j}\neq v_{k} \end{array}}{\sum }\frac{\sigma_{\begin{array}{c} v_{j}\\ v_{k} \end{array}}\left(v_{i}\right)}{\sigma_{v_{j},v_{k}}}$$

where $\sigma_{v_{j},v_{k}}\left(v_{i}\right)$ is the number of shortest paths between node *v_j_* and *v_k_* that pass through node *v_i_*. $\sigma_{v_{j},v_{k}}$ is the total number of shortest paths between *v_j_* and *v_k_*.

We defined the *central point dominance* of each node as:

$$\text{CPD}\left(v_{i}\right)=\frac{1}{N-1}\left(\underset{j}{\max}\left(\text{Betweenness}\left(v_{j}\right)\right)-\text{Betweenness}\left(v_{i}\right)\right)$$

where *N* is the number of nodes in the network.
